# Pollinator-Mediated Selection on Floral Traits of *Primula tibetica* Differs Between Sites With Different Soil Water Contents and Among Different Levels of Nutrient Availability

**DOI:** 10.3389/fpls.2022.807689

**Published:** 2022-03-01

**Authors:** Yun Wu, Xuyu Duan, Zhaoli Tong, Qingjun Li

**Affiliations:** ^1^School of Architecture and Civil Engineering, Xihua University, Chengdu, China; ^2^Yunnan Key Laboratory of Plant Reproductive Adaptation and Evolutionary Ecology, Yunnan University, Kunming, China; ^3^Laboratory of Ecology and Evolutionary Biology, School of Ecology and Environmental Science, Yunnan University, Kunming, China; ^4^College of Landscape Architecture, Sichuan Agricultural University, Chengdu, China

**Keywords:** floral evolution, plant-pollinator interactions, pollinator-mediated selection, *Primula tibetica*, soil N-P-K nutrient availability, soil water content, strength of selection

## Abstract

Abiotic environmental factors are predicted to affect plant traits and the intensity of plant-pollinator interactions. However, knowledge of their potential effects on pollinator-mediated selection on floral traits is still limited. We separately estimated the effects of soil water (two sites with different soil water contents) and N-P-K nutrient availability (different levels of nutrient addition) on pollinator-mediated selection on floral traits of *Primula tibetica* (an insect-pollinated perennial herbaceous species). Our results demonstrated that floral traits, plant reproductive success and pollinator-mediated selection on floral traits varied between sites with different soil water contents and among different levels of nutrient addition. The strength of pollinator-mediated selection was stronger at the site with low soil water content than at the site with high soil water content, and first decreased and then increased with increasing N-P-K nutrient addition. Our results support the hypothesis that abiotic environmental factors influence the importance of pollinators in shaping floral evolution.

## Introduction

One goal of evolutionary ecologists is to understand and predict floral evolutionary responses in complex biotic and abiotic environments (Smith, 2010; Dalrymple et al., 2020). For biotic factors such as pollinators, an increasing number of studies have highlighted their critical role in driving spatial and temporal variation in floral differentiation (Parachnowitsch and Kessler, 2010; Van der Niet et al., 2014; Wu et al., 2018). Pollinators generate selection pressures on many floral traits, including flowering phenology, floral display and traits affecting pollination efficiency (Sandring and Ågren, 2009; Phillips et al., 2017; Sletvold et al., 2017). However, pollinator-mediated selection may be influenced by the abiotic environment (Caruso et al., 2019; Dalrymple et al., 2020; Verloop et al., 2020).

Abiotic environmental factors were shown to affect floral traits (Burkle and Runyon, 2016; Friberg et al., 2017), attractiveness of flowers to pollinators (Maad and Alexandersson, 2004; Glenny et al., 2018) and the reproductive success of the plants (Lau and Stephenson, 1993; Carroll et al., 2001), and cause variation in the intensity of plant-pollinator interactions (Gallagher and Campbell, 2017) and in pollinator-mediated selection on floral traits (Caruso et al., 2005). For example, sulfur deficiency decreased floral display size, caused aberrant flower shapes and led to a clear reduction in flower color and visibility to pollinators (Ausma et al., 2021). Drought stress decreased flower size and flower production (Maad and Alexandersson, 2004; Burkle and Runyon, 2016), decreased nectar volume per flower (Rering et al., 2020), and decreased floral visual traits (Glenny et al., 2018) but increased floral volatile organic compound (VOC) emissions (Campbell et al., 2019). A meta-analysis from Kuppler and Kotowska (2021) demonstrated consistent decreases in floral size, number of flowers and nectar volume in response to reduced water availability. Although Höfer et al. (2021) found that drought did not significantly affect floral volatiles of *Sinapis arvensis* plants, the mean total scent emission per flower tended to be higher in drought-stressed plants than watered plants. Nutrient stress decreased flower production by *Lithophragma bolanderi* (Friberg et al., 2017), whereas soil nutrient addition increased floral display size (Brodbeck et al., 2001). The physiological and biochemical responses of flowers to soil water and nutrient availability may influence pollinator visits and pollination efficiency per visit, thus producing variation in the intensity of plant-pollinator interactions. Soil water and N-P-K nutrient availability may also influence mean population fitness and variance in relative fitness. It is the variance in relative fitness that caps the maximum strength of selection on a given trait (Knight et al., 2006; Benkman, 2013). For example, drought treatment reduced seed production and vegetation biomass of *Polygonum pensylvanicum*, and overall increased variance in relative fitness (Haukos and Smith, 2006). Soil N-P-K nutrient addition increased mean population fitness and reduced variance in relative fitness, thus reducing the strength of selection on floral traits in *Platanthera bifolia* (Mattila and Kuitunen, 2000). Currently, global climate change leads to regional soil water limitations (Burkle and Runyon, 2016), and excessive use of chemical fertilizers in agricultural production is widespread around the world (Yang, 2006; Liu et al., 2015). Under these scenarios, it is necessary to clarify the effect of soil water and nutrient availability (specifically nutrient addition) on the pollination environment, plant-pollinator interactions and pollinator-mediated selection.

Although scientists have recognized the effects of soil water and N-P-K nutrient availability on plant traits and the intensity of plant-pollinator interactions (Caruso et al., 2005; Burkle and Runyon, 2016; Gallagher and Campbell, 2017; Glenny et al., 2018; Campbell et al., 2019), an estimation of the potential role of these abiotic environmental factors in influencing pollinator-mediated selection on floral traits is not well documented. To our knowledge, only one previous study has experimentally quantified the effect of N-P-K nutrient addition on pollinator-mediated selection on floral traits in *Dactylorhiza lapponica*. They found that nutrient addition did not affect flower production, plant reproductive success and pollinator-mediated selection (Sletvold et al., 2017).

To confirm the potential role of soil water and of N-P-K nutrient availability on pollinator-mediated selection, we estimated phenotypic selection on floral traits of *Primula tibetica* at two sites with different soil water contents (low vs. high soil water content) over 2 years, and by manipulating soil N-P-K nutrient availability (four levels of nutrient addition) in a common garden setting. *P. tibetica* is a distylous insect-pollinated perennial herb and is self- and intramorph-incompatible (Jiang and Li, 2017). Our preliminary work showed that soil resource availability influences plant traits in this species. Flowering phenology (i.e., flowering duration per plant) and floral display size (i.e., plant height, number of flowers and corolla size) varied among sites with different soil water content and nutrient availability (Supplementary Figures 1, 2). These variations in floral traits may influence pollinator visitations, the intensity of plant-pollinator interactions and the importance of pollinators in shaping floral evolution. We examined specifically (1) whether pollinator-mediated selection on floral traits differed between sites with different soil water contents and (2) whether the strength of pollinator-mediated selection decreased with increasing N-P-K nutrient availability.

## Materials and Methods

### Study Species

*Primula tibetica* is a distylous insect-pollinated perennial herbaceous species (with long- and short-styled morphs) that is distributed across Tibet, northern India, Nepal and Bhutan along the Himalayas (Richards, 2003). Populations flower from May to August, depending on the elevation, with fruiting from June to August. At our study sites, pollinators of this primrose species include *Bombus richardsi*, a species of *Tachinidae* and a species of *Syrphidae* (Jiang and Li, 2017). Pollinator types (qualitative patterns) did not differ among our study sites (Jiang and Li, 2017).

### Experiment 1: Pollinator-Mediated Selection on Floral Traits at Two Sites With Different Soil Water Contents

#### Field Experiments and Measurements

We conducted experiments at two sites where *P. tibetica* occurred naturally, on SejiLa Mountain, Linzhi County, Xizang, Southwest China (the low soil water content site, 29°46.0325′ N, 94°44.3343′ E, 3,353 m.a.sl. and the high soil water content site, 29°46.0459′ N, 94°44.1721′ E, 3,351 m.a.sl.). These two sites were approximately 150 m apart. From June 7 to August 22 in 2019 and 2020, we measured the soil moisture content at each site every 15 days. For each measurement, we randomly selected 10 spots and measured the soil moisture content for each spot at each site. We estimated the mean soil moisture content per spot (totally 10 spots) for each measurement at each site. The soil moisture content ranged from 33 to 59% (mean ± SD = 43.3 ± 9.5) and from 23 to 44% (mean ± SD = 29.3 ± 8.5) for the high and low soil water content sites, respectively. Soil total nitrogen [*F*_(1, 19)_ = 0.095, *P* = 0.761], total potassium [*F*_(1, 19)_ = 1.359, *P* = 0.259] and total phosphorus [*F*_(1, 19)_ = 0.0005, *P* = 0.983] did not differ between the sites (Supplementary Figure 3).

In 2019, we randomly chose and marked 200 and 220 individuals at the sites with low and high soil water contents, respectively. In 2020, we randomly chose and marked 240 and 260 individuals at the sites with low and high soil water contents, respectively. At each site, we assigned these individuals to one of two treatments: open pollination (C) or supplemental hand pollination (HP). We pollinated the long-styled morph flowers with short-styled morph pollen and the short-styled morph flowers with long-styled morph pollen. Because the flowering duration of a single flower was approximately 6–7 days (*own field observations*), we visited these individuals every 4 days throughout the flowering period. At each visit, all new open flowers in the HP treatment were pollinated by hand with pollen from other individuals located at least 10 m from the target individual. All flowers in the HP treatment received hand pollination at least once.

We recorded the start and end of flowering (Julian day) for each individual as the day when the first flower opened and the last flower wilted, respectively. We calculated the flowering duration for each individual as the end of flowering minus the start of flowering. At the onset of flowering, we measured the plant height of each individual (distance from the ground to the topmost flower to the nearest 0.1 cm). For the first three open flowers on each individual, we measured corolla size (maximum diameter of the corolla), corolla tube size (maximum diameter of the corolla tube entrance) and corolla tube length (distance from the corolla tube entrance to the bottom of the corolla tube) to the nearest 0.01 mm with digital calipers. In some individuals only one or two flowers could be measured as the individuals did not produce more flowers. We calculated the mean (if more than one flower was available) corolla size, corolla tube size and corolla tube length for each individual. We recorded the number of flowers for each individual at the end of the flowering period.

To quantify reproductive success, we recorded the number of fruits at maturation, and these fruits were collected to determine the number of seeds per fruit. The total seed production per plant was then calculated for all sampled plants.

For each year and site, we quantified the degree of pollen limitation (PL) as 1 - (mean total seed production per plant of C plants/mean total seed production per plant of HP plants). We calculated 95% confidence intervals (CIs) for pollen limitation using bootstrapping (2,000 iterations; boot package in R; Canty and Ripley, 2013).

#### Statistical Analysis

We tested the effects of year, site, pollination (C vs. HP) and their interactions on each floral trait (flowering start date, flowering end date, flowering duration, plant height, number of flowers, corolla size, corolla tube size and corolla tube length) and reproductive success (fruit production, seeds per fruit and seeds per plant) using multiple three-way analysis of variance (ANOVA) models. To achieve a normal distribution, we log_10_ transformed the data prior to ANOVA (variances of data were homogenous).

Following the methods of Lande and Arnold (1983), we estimated directional (β_i_), and stabilizing or disruptive (γ_ii_) selection gradients from multiple linear and non-linear regression models, respectively, separately for each pollination treatment, site and year. Both models used the relative number of seeds per plant as the response variable. In the linear regression models, we used the six standardized floral traits (flowering duration, plant height, number of flowers, corolla size, corolla tube size and corolla tube length) as the explanatory variables. In the non-linear regression models, we used both the linear and quadratic terms of the six standardized floral traits (as mentioned above) as the explanatory variables. For each pollination treatment, site and year, we calculated the relative number of seeds per plant by dividing the individual seeds per plant by the treatment mean, and trait values were standardized to a mean of 0 and a variance of 1 (both using the original data). For each non-linear regression model, we calculated stabilizing or disruptive selection gradients by multiplying the quadratic regression coefficients by 2 (Stinchcombe et al., 2008). To test for multicollinearity in these linear and non-linear regression models, we calculated variance inflation factors (VIFs) for the linear and non-linear terms. All VIFs for the linear and non-linear terms were < 5.0, indicating that multicollinearity was sufficiently low and did not have a large impact on model coefficients (Quinn and Keough, 2002).

To quantify pollinator-mediated selection, we subtracted the estimated selection gradients of each trait for plants that were subjected to the HP treatment (β_HP_ or γ_HP_) from the estimates obtained for plants under the C treatment (β_C_ or γ_C_) (Δβ_poll_ = β_C_–β_HP_, Δγ_poll_ = γ_C_–γ_HP_) with its associated standard error $\sqrt{S\,E_{\beta_{C}}^{2}+S\,E_{\beta_{H\,P}}^{2}}$ or $\sqrt{S\,E_{\gamma_{C}}^{2}+S\,E_{\gamma_{H\,P}}^{2}}$ (Chapurlat et al., 2019). To test whether pollinator-mediated selection varied between sites or between years, we included the data from plants in both the C and HP treatments at the two sites and in the 2 years using ANCOVA. The model included the relative number of seeds per plant as the response variable and the six standardized traits, site, year, pollination (C vs. HP), and their interactions (i.e., trait × site, trait × year, trait × pollination, trait × site × year, trait × site × pollination, trait × year × pollination and trait × site × year × pollination) as explanatory variables. Significant trait × site × pollination interactions indicated that pollinator-mediated selection varied between sites. Significant trait × year × pollination interactions indicated that pollinator-mediated selection varied between years. Because some trait × site × year × pollination interactions were significant, we further tested whether pollinator-mediated selection varied between sites separately for each year.

To test whether the strength of net selection and the strength of pollinator-mediated selection varied between sites and/or between years, we analyzed the absolute values of net selection gradients (|β_C_| or |γ_C_|) and pollinator-mediated selection gradients (|Δβ_poll_| or |Δγ_poll_|) of the six traits using ANCOVA. The model included the absolute values of net selection gradients or the absolute values of pollinator-mediated selection gradients as the response variable and site, year and the site × year interaction as the explanatory variables.

### Experiment 2: Pollinator-Mediated Selection on Floral Traits Among Different Levels of N-P-K Nutrient Addition

#### Field Experiments and Measurements

We conducted this experiment at the South-East Tibetan Plateau Station for Integrated Observation and Research of Alpine Environment, Chinese Academy of Sciences (29°46′ N, 94°44′ E, 3,326 m.a.sl.). At the study site, natural pollinators of the plant were present. The pollinator community (qualitative patterns) included *Bombus richardsi*, a species of *Tachinidae* and a species of *Syrphidae*.

During early May 2020, we chose and marked 864 separate individuals of *P. tibetica*. These individuals were collected from the high soil water content site (29°46.0459′ N, 94°44.1721′ E, 3,351 m.a.sl.) and had not grown a scape at this time. We transplanted these plants into polythene plastic pots (length × width × height was 9 × 9 × 6 cm; Garden Flowers Company, Suqian, CN) with one individual per pot. The pot was filled with a soil matrix, that included perlite, vermiculite and coco coir (at a volume ratio of 1:1.5:1). After transplanting, we added water (50 ml) to each pot and then exposed these plants to the environment. We provided water every 3 days to train the plants, and no plants died.

Two weeks later, we randomly assigned these individuals to one of eight treatments (108 individuals per treatment) in a factorial design for pollination (C and HP) vs. N-P-K nutrient addition (four levels of nutrient availability). The treatments were as follows: open pollination, no N-P-K nutrient addition (C0N); supplemental hand pollination, no N-P-K nutrient addition (HP0N); open pollination, 0.5% (weight/weight) N-P-K nutrient addition (C05N); supplemental hand pollination, 0.5% N-P-K nutrient addition (HP05N); open pollination, 1% N-P-K nutrient addition (C1N); supplemental hand pollination, 1% N-P-K nutrient addition (HP1N); open pollination, 2% N-P-K nutrient addition (C2N); and supplemental hand pollination, 2% N-P-K nutrient addition (HP2N). We used N-P-K fixed fertilizer (NPK20-20-20+TE microelement, Beijing Bowei Shennong Technology Co., Ltd., Beijing, CN) and diluted it to solutions of different concentrations (Supplementary Table 1). Nitrogen was supplied as both nitrate and ammonium. The range of N-P-K nutrient addition concentrations was based on preliminary experiments (*own observations*), which demonstrated that less than 2% N-P-K nutrient concentrations were ecologically relevant at the study sites. For each individual in a nutrient addition treatment, we added 50 ml of N-P-K compound solution. For each individual in no nutrient addition treatment, we added 50 ml of water. To limit the effect of individual distribution on pollinator visitations and pollinator-mediated selection, we randomly assigned all individuals within the common garden setting. All individuals were exposed to the natural environment.

Plants began to flower approximately 20 d after the first N-P-K nutrient addition treatment. We conducted the nutrient additions for each individual every 15 days throughout the flowering period. Because most individuals in the C2N (85%) and HP2N (83%) treatments died before fruit ripening (continuous addition of a high concentration of nutrients damaged the roots of plants, resulting in wilting and then mortality), we collected data only on plants from the C0N, HP0N, C05N, HP05N, C1N and HP1N treatments. We visited the HP plants every 4 days throughout the flowering period to conduct hand pollination. At each visit, all new open flowers on plants in the HP treatment were pollinated by hand with fresh pollen, following the methods of Experiment 1. Some plants did not produce flowers. In the experiment, the distribution of flowering/non-flowering plants was random within the common garden setting. The number of plants that died in the C0N, HP0N, C05N, HP05N, C1N, and HP1N treatments was 12, 15, 23, 20, 30, and 37, respectively.

Following the methods of Experiment 1, we recorded the flowering start date, flowering end date, flowering duration, plant height, number of flowers, corolla tube size, corolla tube length, fruit production, seeds per fruit and seeds per plant for each individual. For each nutrient addition treatment, we quantified the degree of PL and its 95% confidence intervals (CIs) as above.

#### Statistical Analysis

We tested the effects of nutrient addition, pollination (C vs. HP) and their interactions on each floral trait (flowering start date, flowering end date, flowering duration, plant height, number of flowers, corolla tube size and corolla tube length) and reproductive success (fruit production, seeds per fruit and seeds per plant) using multiple two-way ANOVA models. To achieve a normal distribution, we log_10_ transformed the data prior to ANOVA (variances of data were homogenous).

Following the methods of Experiment 1, we estimated directional and stabilizing or disruptive selection gradients (β_i_ or γ_ii_) from multiple linear and non-linear regression models, respectively, separately for each treatment. Both models used the relative number of seeds per plant as the response variable. In the linear regression models, we used the six standardized floral traits (flowering start date, flowering duration, plant height, number of flowers, corolla tube size and corolla tube length) as the explanatory variables. In the non-linear regression models, we used both the linear and quadratic terms of the six standardized floral traits (as mentioned above) as the explanatory variables. All VIFs of the linear and non-linear terms were < 5.1, indicating that multicollinearity was sufficiently low and did not have a large impact on model coefficients (Quinn and Keough, 2002).

We quantified pollinator-mediated selection (Δβ_poll_ = β_C_–β_HP_ or Δγ_poll_ = γ_C_–γ_HP_) with its associated standard error for each nutrient addition treatment following the methods of Experiment 1. To test whether pollinator-mediated selection varied among nutrient addition treatments, we included data from plants in both the C and HP treatments at three levels of nutrient addition in ANCOVA. The model included the relative number of seeds per plant as the response variable and the six standardized traits, nutrient addition, pollination (C vs. HP), trait × nutrient addition, trait × pollination and trait × nutrient addition × pollination as the explanatory variables. Significant trait × nutrient addition × pollination interactions indicated that pollinator-mediated selection varied among nutrient addition treatments.

To estimate whether the strength of net selection and the strength of pollinator-mediated selection varied among nutrient addition treatments, we used ANCOVA following the methods of Experiment 1. The ANCOVA model included the absolute values of net selection gradients or absolute values of pollinator-mediated selection gradients as the response variable and nutrient addition (three levels) as the explanatory variable. We performed all analyses with R 3.6.3 (R Core Team, 2019).

## Results

### Experiment 1: Floral Traits and Reproductive Success

Floral traits differed between years (except for the flowering start date) and sites (*P* < 0.05; Tables 1, 2). In 2019, the flowering start date of the HP plants at the high soil water content site was earlier than that at the low soil water content site. The opposite trend was observed in 2020. For the C plants, the flowering start date was the same at the two sites in 2019 and was later at the high soil water content site than at the low soil water content site in 2020. Plants at the high soil water content site had a later (by 2.7% in 2019, 2.8% in 2020) flowering start date, a longer (by 31.8, 7.8%) flowering duration, a taller (by 18.3, 24.5%) plant height, a greater (by 56.6, 35.7%) number of flowers, a larger (by 11.9, 7.3%) corolla size, a larger (by 0.3, 5.2%) corolla tube size and a shorter (by 8.4, 2.3%) corolla tube length than plants at sites with low soil water content (*P* < 0.05 each, N2019 = 326, N2020 = 354).

**TABLE 1:** ANOVA tests of the effect of year, site, pollination treatment and their interactions on floral traits and reproductive success of *Primula tibetica*.

Traits and reproductive success	Year		Site		Pollination		Year × site		Year × pollination		Site × pollination		Year × site × pollination	
							
	*F*_(1, 679)_	*P*	*F*_(1, 679)_	*P*	*F*_(1, 679)_	*P*	*F*_(1, 679)_	*P*	*F*_(1, 679)_	*P*	*F*_(1, 679)_	*P*	*F*_(1, 679)_	*P*
Flowering start date	0.508	0.476	5.582	**0.018**	0.205	0.651	23.903	**< 0.001**	0.781	0.377	2.499	0.114	0.848	0.357
Flowering end date	25.044	**< 0.001**	78.442	**< 0.001**	4.467	**0.035**	0.004	0.953	2.482	0.116	4.093	**0.044**	1.259	0.262
Flowering duration	21.546	**< 0.001**	64.778	**< 0.001**	3.829	0.051	24.268	**< 0.001**	0.147	0.701	0.822	0.365	0.965	0.326
Plant height	152.44	**< 0.001**	87.431	**< 0.001**	0.045	0.832	2.413	0.121	1.694	0.194	1.283	0.258	5.484	**0.02**
Number of flowers	118.959	**< 0.001**	136.754	**< 0.001**	1.562	0.212	6.851	**0.009**	1.787	0.182	0.119	0.731	2.119	0.146
Corolla size	125.872	**< 0.001**	113.603	**< 0.001**	3.43	0.065	4.865	**0.028**	0.309	0.578	0.309	0.578	0.512	0.475
Corolla tube size	157.663	**< 0.001**	8.236	**0.004**	1.213	0.271	5.274	**0.022**	2.771	0.096	10.532	**0.001**	1.804	0.18
Corolla tube length	30.46	**< 0.001**	23.24	**< 0.001**	0.074	0.786	11.817	**< 0.001**	1.261	0.262	1.234	0.267	0.781	0.377
Fruit production	119.008	**< 0.001**	150.044	**< 0.001**	98.675	**< 0.001**	7.567	**0.006**	0.082	0.775	0.179	0.672	0.364	0.546
Seeds per fruit	36.618	**< 0.001**	120.971	**< 0.001**	207.31	**< 0.001**	8.156	**0.004**	15.524	**< 0.001**	4.964	**0.026**	7.464	**0.006**
Seeds per plant	96.824	**< 0.001**	195.321	**< 0.001**	229.468	**< 0.001**	11.609	**< 0.001**	8.357	**0.004**	1.777	0.183	2.53	0.112

*Bold P-values indicate significant effects (at the significance level of 0.05).*

**TABLE 2:** Floral traits and reproductive success (mean ± SD) of *Primula tibetica* in 2019 and 2020.

Traits and reproductive success	High soil water content site 2019		Low soil water content site 2019		High soil water content site 2020		Low soil water content site 2020	
	C (*n* = 89)	HP (*n* = 96)	C (*n* = 70)	HP (*n* = 71)	C (*n* = 93)	HP (*n* = 88)	C (*n* = 86)	HP (*n* = 87)
Flowering start date (Julian day)	153.4 ± 2.4	152.3 ± 5.0	153.5 ± 4.2	154.6 ± 1.9	154.6 ± 8.0	154.9 ± 7.6	151.0 ± 6.9	152.0 ± 8.1
Flowering end date (Julian day)	177.7 ± 4.3	176.5 ± 3.7	171.4 ± 6.8	173.5 ± 6.2	174.2 ± 8.7	175.6 ± 8.3	169.0 ± 7.3	171.3 ± 7.7
Flowering duration (d)	24.3 ± 4.8	24.2 ± 5.0	17.9 ± 6.9	18.9 ± 5.9	19.5 ± 5.8	20.6 ± 6.3	17.9 ± 5.2	19.3 ± 7.0
Plant height (cm)	4.2 ± 1.1	4.2 ± 1.1	3.7 ± 1.4	3.4 ± 1.1	3.4 ± 1.1	3.2 ± 0.9	2.5 ± 1.2	2.8 ± 1.1
Number of flowers (per plant)	4.2 ± 1.1	4.1 ± 1.4	2.5 ± 1.1	2.8 ± 1.6	2.7 ± 1.3	3.0 ± 1.2	2.1 ± 1.2	2.1 ± 1.0
Corolla size (mm)	12.35 ± 1.08	12.62 ± 1.20	11.19 ± 1.47	11.13 ± 1.18	11.07 ± 1.13	11.28 ± 1.12	10.28 ± 1.34	10.54 ± 1.53
Corolla tube size (mm)	1.64 ± 0.23	1.55 ± 0.17	1.55 ± 0.25	1.63 ± 0.19	1.42 ± 0.18	1.43 ± 0.19	1.32 ± 0.21	1.39 ± 0.21
Corolla tube length (mm)	4.39 ± 0.49	4.35 ± 0.54	4.78 ± 0.57	4.76 ± 0.62	4.78 ± 0.61	4.74 ± 0.50	4.80 ± 0.83	4.94 ± 0.73
Fruit production (per plant)	3.0 ± 1.2	4.0 ± 1.4	1.8 ± 0.8	2.6 ± 1.2	1.9 ± 1.0	2.8 ± 1.1	1.3 ± 0.9	2.0 ± 0.8
Seeds per fruit (per plant)	17.4 ± 6.8	27.1 ± 8.8	10.0 ± 4.5	14.5 ± 5.7	12.8 ± 7.0	20.2 ± 6.2	8.0 ± 5.7	17.5 ± 5.7
Seeds per plant	56.4 ± 39.4	114.4 ± 70.0	19.2 ± 12.9	38.7 ± 28.4	25.9 ± 18.5	58.1 ± 32.3	13.1 ± 13.2	35.7 ± 21.3

*C, open pollination treatment; HP, supplemental hand pollination treatment. Sample size (n) is given. All floral traits (except for flowering start date) and reproductive success significantly differed between years and between sites. Reproductive success significantly varied between pollination treatments.*

Fruit production, seeds per fruit and seeds per plant differed between years, sites and pollination treatments (Table 2). Plants at the site with high soil water content produced more fruit, more seeds per fruit and a higher number of seeds per plant than those at the site with low soil water content (Table 1). The HP treatment increased reproductive success compared with the C treatment.

In 2019, the PL indices were 0.507 (lower and upper CIs, 0.407 and 0.591) and 0.504 (0.373 and 0.608) at sites with high and low soil water contents, respectively. In 2020, the PL indices were 0.554 (0.466 and 0.628) and 0.633 (0.53 and 0.715) at sites with high and low soil water contents, respectively.

### Experiment 1: Phenotypic Selection

**FIGURE 1:**
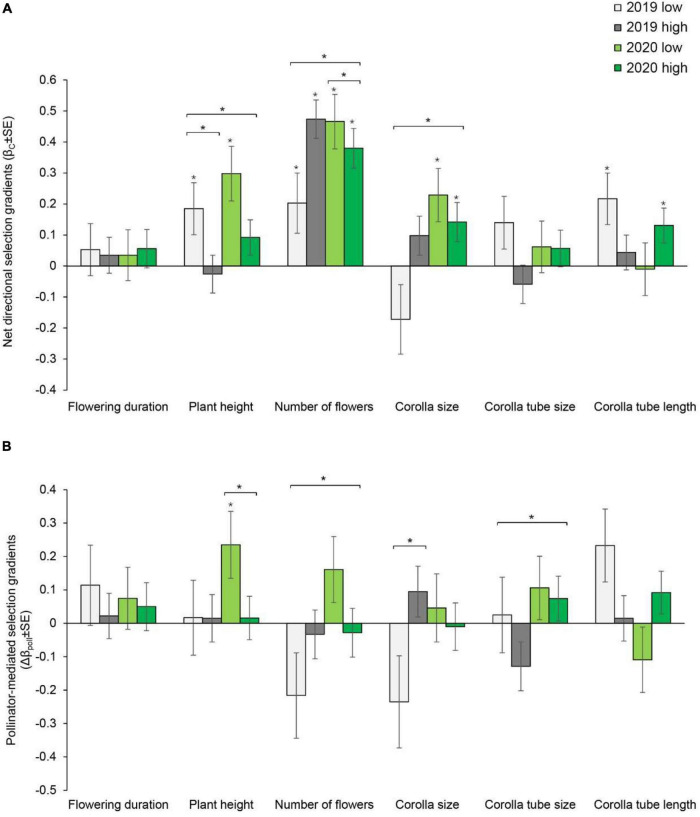
Net directional selection gradients **(A)** and pollinator-mediated selection gradients **(B)** on flowering duration, plant height, number of flowers, corolla size, and corolla tube size and length in *Primula tibetica* at two sites (low and high soil water content sites). The symbols above the individual bars indicate the level of significance of the selection gradient. Symbols above the short lines spanning two gradients show whether ANCOVA indicated significant variation between sites in each year. Symbols above the long lines spanning several gradients show whether ANCOVA indicated significant variation between years. *P < 0.05.

**FIGURE 2:**
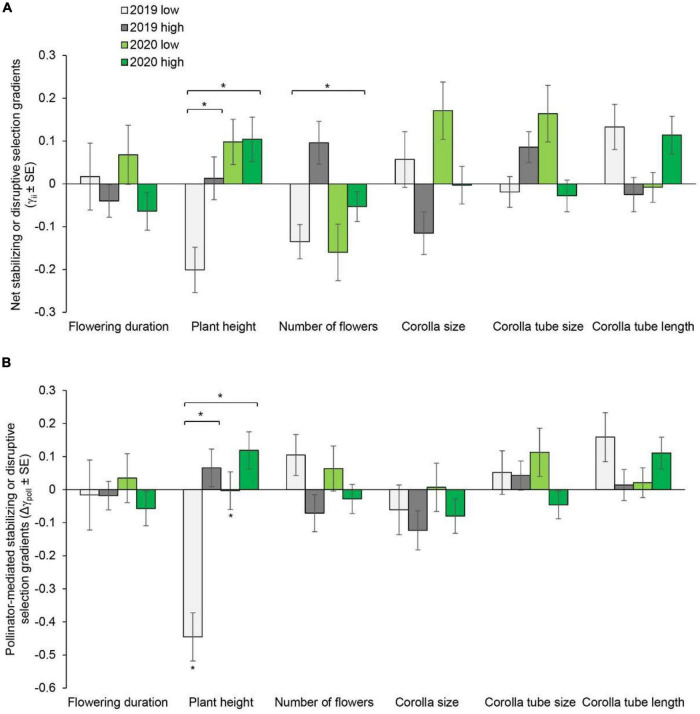
Net stabilizing or disruptive selection gradients **(A)** and pollinator-mediated selection gradients **(B)** on flowering duration, plant height, number of flowers, corolla size, and corolla tube size and length in *Primula tibetica* at two sites (low and high soil water content sites). The symbols above the individual bars indicate the level of significance of the selection gradient. Symbols above the short lines spanning two gradients show whether ANCOVA indicated significant variation between sites in each year. Symbols above the long lines spanning several gradients show whether ANCOVA indicated significant variation between years. *P < 0.05.

In 2019, net directional selection for taller plants, a greater number of flowers and a longer corolla tube was detected at the site with low soil water content. In 2020, net directional selection for taller plants, a greater number of flowers and larger corolla size was detected (Supplementary Table 2 and Figure 1A). At the site with high soil water content, selection for a greater number of flowers was detected in both years. In addition, selection for larger corolla size and a longer corolla tube was detected in 2020 (Supplementary Table 2 and Figure 1A). The net directional selection on plant height in 2019 [*F*_(1, 178)_ = 11.559, *P* < 0.001] and on the number of flowers in 2020 [*F*_(1, 158_) = 6.623, *P* = 0.011] varied between sites (Supplementary Table 3). Significant net stabilizing or disruptive selection was not detected for any floral traits (Supplementary Table 4 and Figure 2A).

Pollinator-mediated selection on the number of flowers and corolla tube size varied between years, as indicated by the significant year × pollination × number of flowers interaction [*F*_(1, 679)_ = 8.069, *P* = 0.005] and year × pollination × corolla tube size interaction [*F*_(1, 679)_ = 4.748, *P* = 0.03] (Supplementary Table 5). At the site with low soil water content, pollinator-mediated selection for taller plant height was detected in 2020 (Supplementary Table 2, Supplementary Figure 4, and Figure 1B). Pollinator-mediated stabilizing selection on plant height was detected in both years at the site with low soil water content (Supplementary Table 4 and Figure 2B). Pollinator-mediated stabilizing selection on plant height varied between years [*F*_(1, 679)_ = 5.331, *P* = 0.021; Supplementary Table 6] and between sites in 2019 [*F*_(1, 325)_ = 6.497, *P* = 0.011; Supplementary Table 7].

**FIGURE 3:**
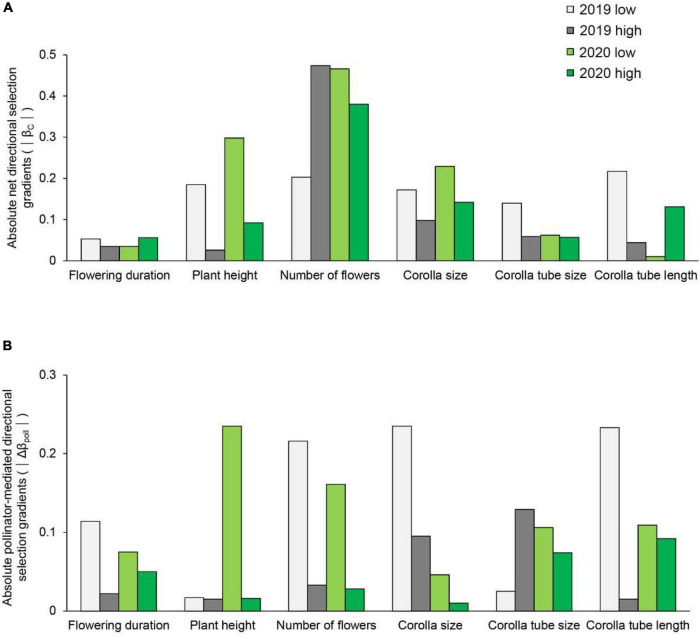
The strength of net directional selection **(A)** and pollinator-mediated selection **(B)** on flowering duration, plant height, number of flowers, corolla size, and corolla tube size and length in *Primula tibetica* at two sites (low and high soil water content sites).

The strength of net directional selection and stabilizing or disruptive selection on floral traits did not vary between years or between sites (Supplementary Table 8); however, the strength of pollinator-mediated directional selection varied between sites [*F*_(1, 23)_ = 8.894, *P* = 0.007; Supplementary Table 8]. The strength of pollinator-mediated directional selection was stronger at the site with low soil water content than at the site with high soil water content (except for corolla tube size in 2019; Figure 3). The strength of pollinator-mediated directional selection on flowering duration, number of flowers, corolla size and corolla tube length were stronger at the site with low soil water content in both years. The strength of pollinator-mediated stabilizing or disruptive selection on floral traits did not vary between years or between sites (Supplementary Table 8).

### Experiment 2: Floral Traits and Reproductive Success

The flowering start date, flowering end date, plant height, corolla tube size and corolla tube length did not vary among nutrient addition treatments or between pollination treatments (*P* > 0.05; Table 3). Flowering duration and the number of flowers differed among nutrient addition treatments (Table 3). For the C plants, flowering duration increased with increasing nutrient addition; however, plants produced more flowers in both the C05N and C1N treatments. For the HP plants, flowering duration and the number of flowers first increased and then decreased with increasing nutrient availability.

**TABLE 3:** Floral traits and reproductive success (mean ± SD) of *Primula tibetica* in each treatment.

Traits and reproductive success	0% nutrient addition		0.5% nutrient addition		1% nutrient addition		Nutrient addition		Pollination		Nutrient addition × pollination	
						
	C (*n* = 73)	HP (*n* = 62)	C (*n* = 47)	HP (*n* = 64)	C (*n* = 53)	HP (*n* = 59)	*F*_(2, 357)_	*P*	*F*_(1, 357)_	*P*	*F*_(2, 357)_	*P*
Flowering start date (Julian day)	174.9 ± 7.5	174.0 ± 5.7	173.5 ± 5.6	173.2 ± 5.3	172.8 ± 6.2	173.4 ± 4.9	1.76	0.174	0.121	0.729	0.397	0.672
Flowering end date (Julian day)	193.6 ± 6.2	193.9 ± 5.8	195.0 ± 5.1	195.4 ± 5.9	194.7 ± 6.7	194.2 ± 5.1	2.076	0.127	0.023	0.879	0.182	0.833
Flowering duration (d)	18.7 ± 5.6	19.9 ± 5.7	21.5 ± 6.1	22.3 ± 6.0	21.8 ± 5.0	20.8 ± 5.5	**7.532**	**< 0.001**	0.32	0.572	1.518	0.221
Plant height (cm)	4.3 ± 1.7	3.9 ± 1.7	3.9 ± 1.5	4.2 ± 1.6	4.2 ± 1.4	4.2 ± 1.6	0.977	0.378	0.273	0.601	0.882	0.415
Number of flowers (per plant)	3.0 ± 1.4	3.5 ± 1.5	3.9 ± 1.6	4.5 ± 1.6	3.9 ± 1.2	3.8 ± 1.3	**14.098**	**< 0.001**	**4.403**	**0.037**	2.735	0.066
Corolla tube size (mm)	1.44 ± 0.20	1.51 ± 0.16	1.51 ± 0.16	1.49 ± 0.15	1.49 ± 0.21	1.51 ± 0.14	1.256	0.286	3.638	0.057	2.523	0.082
Corolla tube length (mm)	5.57 ± 0.59	5.62 ± 0.44	5.56 ± 0.53	5.48 ± 0.42	5.41 ± 0.53	5.59 ± 0.51	1.123	0.327	1.366	0.243	1.691	0.186
Fruit production (per plant)	1.7 ± 1.1	2.7 ± 1.5	2.6 ± 1.6	3.1 ± 1.8	1.4 ± 1.2	2.2 ± 1.3	**13.017**	**< 0.001**	**21.725**	**< 0.001**	1.662	0.191
Seeds per fruit (per plant)	17.2 ± 12.4	26.1 ± 12.4	22.7 ± 11.8	23.9 ± 12.2	12.8 ± 10.7	16.2 ± 12.4	**11.771**	**< 0.001**	**11.549**	**< 0.001**	1.364	0.257
Seeds per plant	35.1 ± 32.7	79.4 ± 58.6	67.5 ± 47.9	81.4 ± 60.8	25.7 ± 29.4	41.0 ± 43.5	**15.076**	**< 0.001**	**19.038**	**< 0.001**	1.883	0.154

*C, open pollination treatment; HP, supplemental hand pollination treatment. Sample size (n) is given.*

*Bold P-values indicate significant effects (at the significance level of 0.05).*

Reproductive success differed among nutrient addition treatments and between pollination treatments (Table 3). In the C plants, the highest fruit production, highest number of seeds per fruit and highest number of seeds per plant was observed in the 0.5% N-P-K nutrient addition treatment. This was followed by the 0% N-P-K nutrient addition treatment. The lowest reproductive success was observed in the 1% N-P-K nutrient addition treatment. The HP treatment increased fruit production, seeds per fruit and seeds per plant compared with the C treatment (Table 3).

The PL indices were 0.558 (lower and upper CIs, 0.414 and 0.667), 0.171 (–0.083 and 0.374) and 0.373 (0.059 and 0.586) in the 0, 0.5, and 1% N-P-K nutrient addition treatments, respectively.

### Experiment 2: Phenotypic Selection

**FIGURE 4:**
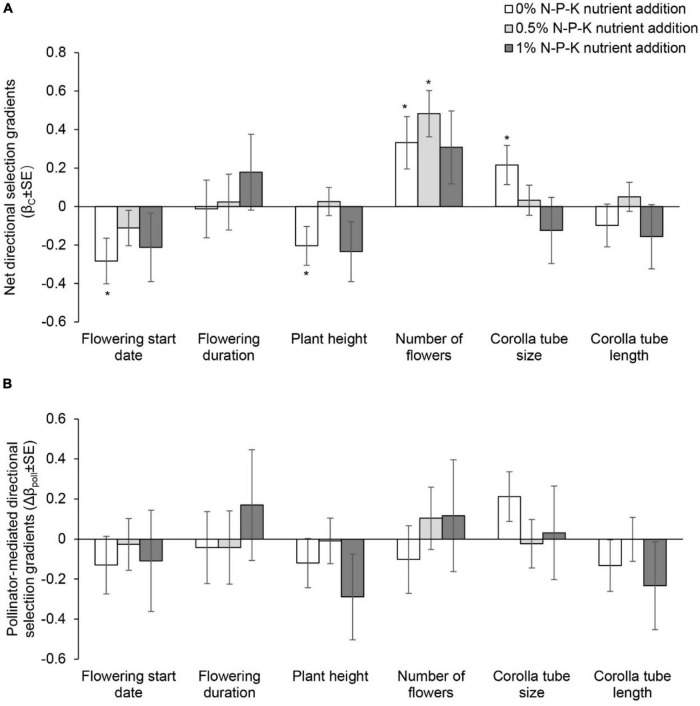
Net directional selection gradients **(A)** and pollinator-mediated selection gradients **(B)** on flowering start date, flowering duration, plant height, number of flowers, and corolla tube size and length in *Primula tibetica* among nutrient addition treatments. The symbols above the individual bars indicate the level of significance of the selection gradient. Net selection and pollinator-mediated selection on floral traits did not vary among nutrient addition treatments (*P* > 0.05; Supplementary Tables 14, 15). *P < 0.05.

**FIGURE 5:**
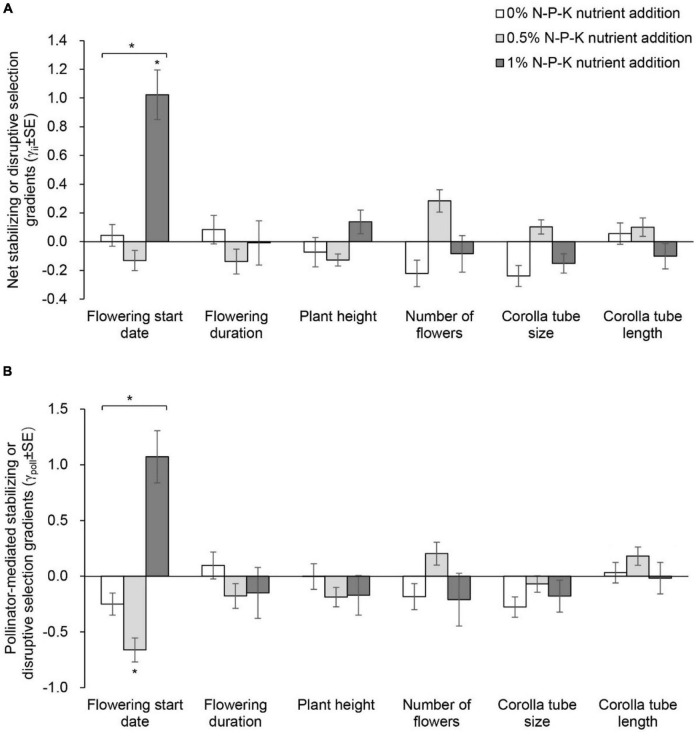
Net stabilizing or disruptive selection gradients **(A)** and pollinator-mediated selection gradients **(B)** on flowering start date, flowering duration, plant height, number of flowers, and corolla tube size and length in *Primula tibetica* among nutrient addition treatments. The symbols above the individual bars indicate the level of significance of the selection gradient. Symbols above the lines spanning several gradients show whether ANCOVA indicated significant variation among nutrient addition treatments in selection gradients. *P < 0.05.

In the C0N treatment, net directional selection for an earlier flowering start date, shorter plant height, a greater number of flowers and larger corolla tube size was detected (Supplementary Table 9 and Figure 4A). In the C05N treatment, net directional selection for a greater number of flowers was detected (Figure 4A). In the C1N treatment, net directional selection was not detected for any floral traits. The strength of net directional selection did not vary among nutrient addition treatments (Supplementary Table 10). In the C1N treatment, net disruptive selection for flowering start date was detected (Supplementary Table 11 and Figure 5A). The strength of net stabilizing or disruptive selection on floral traits did not vary among nutrient addition treatments (Supplementary Table 10).

The strength of pollinator-mediated directional selection on floral traits differed among nutrient addition treatments [*F*_(2, 17)_ = 5.634, *P* = 0.015; Supplementary Tables 10, 12, and Figure 4B]. The strength of selection at the low and high levels of nutrient addition was stronger than that at the intermediate level of nutrient addition. Specifically, the strength of pollinator-mediated directional selection on flowering start date, plant height, corolla tube size and corolla tube length was weakest at the intermediate level of nutrient addition. Pollinator-mediated stabilizing selection on flowering start date occurred in the 0.5% N-P-K nutrient addition treatment (Supplementary Table 13 and Figure 5B). The strength of pollinator-mediated stabilizing or disruptive selection on floral traits did not vary among nutrient addition treatments (Supplementary Table 10).

## Discussion

Our studies demonstrated net directional selection on floral display traits (including plant height and number of flowers) of *P. tibetica* in both experiments. Pollinators mediated directional and stabilizing selection on plant height at the site with low soil water content. Pollinator-mediated stabilizing selection on flowering start date occurred in the 0.5% N-P-K nutrient addition treatment. Furthermore, the strength of pollinator-mediated directional selection on floral traits differed between sites that differed in soil water content. The strength of selection was stronger at the site with low soil water content than at the site with high soil water content, and first decreased and then increased with increasing nutrient availability. Our results support the hypothesis that abiotic environmental factors influence the importance of pollinators in shaping floral evolution.

### Pollinator-Mediated Selection on Floral Traits at Two Sites With Different Soil Water Contents

Previous studies have demonstrated that taller plants are selected by pollinators because taller plants are easily recognized (Ågren et al., 2006; Sletvold and Ågren, 2010). Our results demonstrated that pollinators simultaneously mediated directional selection for taller plants and stabilizing selection on plant height at the site with low soil water content. Thus, tall and intermediate plants seem to have higher plant fitness benefits than small plants.

Previous studies have indicated a role for pollinators in driving positive selection on floral display traits because these traits influence the attractiveness of flowers to pollinators (Grindeland et al., 2005; Sletvold and Ågren, 2010). However, our results showed that pollinators mediated marginally significant selection for less flower production and smaller corolla size at the site with low soil water content in 2019. This finding might be related to the evolution of self-fertilization at the low soil water content site. Self-fertilized plants usually produce small flowers and fewer rewards, whereas cross-fertilized plants preferentially produce large, showy flowers to attract pollinators (Jiang and Li, 2017). In *Primula*, the evolution of self-fertilization from cross-fertilization was accompanied by smaller and less conspicuous flowers (Vos et al., 2014). In this scenario, pollinators may have no choice to select greater/larger flowers. Marginally significant pollinator-mediated selection for fewer/smaller flowers may be a result of the evolution of self-fertilization from cross-fertilization. Unfortunately, we cannot completely explain this result until manipulative experiments are conducted. Furthermore, our results demonstrated that pollinator-mediated selection on the number of flowers and corolla tube size varied between years at both sites, especially the direction of selection. This suggests that pollinator-mediated selection is not consistent on the time scales of these experiments. At our study sites, the soil moisture content varied between years. Soil moisture content may influence plant development, pollinator visitations and the intensity of plant-pollinator interactions. Thus, temporal variations in soil moisture may contribute to the year-to-year differences in selection on floral traits.

Our results demonstrated that the strength of pollinator-mediated selection on floral traits varied between the two sites with different soil water contents. The strength of selection was stronger at the site with low soil water content than at the site with high soil water content. Soil water availability influences plant traits, thus influencing pollinator visitation rates (Burkle and Runyon, 2016; Gallagher and Campbell, 2017; Campbell et al., 2019). Our results indicated that plants were shorter, had less flower production and had smaller corollas at the site with low soil water content. This may have reduced the attractiveness of flowers to pollinators. As a result, pollen limitation was stronger at the site with low soil water content, resulting in stronger pollinator-mediated selection on floral traits.

Our results demonstrated net selection on plant height, number of flowers, corolla size and corolla tube length. However, pollinator-mediated selection was only detected for plant height. This suggests that non-pollinator agents also shape the floral evolution of *P. tibetica*. Indeed, our results demonstrated that non-pollinator agents mediated directional selection for taller plants, greater flower production, larger corolla size and a longer corolla tube length at the study sites. Abiotic factors, such as soil resource availability, affect floral development and growth (Burkle and Runyon, 2016; Campbell et al., 2019). These factors will influence natural selection. Our results indicated variations in net directional selection on plant height and number of flowers between sites with different soil water contents. This suggests that soil water availability may be one of the abiotic factors that contribute to selection on these traits. Non-pollinator agents-mediated selection on corolla size was only detected in one study year. In addition, net directional selection on corolla tube length in natural populations was separately demonstrated at the site with low soil water content in 2019 and at the site with high soil water content in 2020. This suggests that other unknown abiotic factors may also generate selection pressures on these two traits. In the present study, we did not conduct specific experiments to exclude the effect of other factors (i.e., genetic make-up of the populations, interspecies competition or other environmental stress differences between the sites) on trait differences and reproductive success between sites. These factors can also contribute to spatial and temporal variation in selection on floral traits (Caruso et al., 2019; Wu et al., 2021).

### Pollinator-Mediated Selection on Floral Traits Among Different Levels of N-P-K Nutrient Addition

In our common garden experiments with three levels of nutrient addition, significant pollinator-mediated directional selection on floral traits was not detected. However, pollinator-mediated stabilizing selection on flowering start date was detected in the 0.5% N-P-K nutrient addition treatment. This suggested that plants with an intermediate flowering start date would be selected by pollinators. In this case, the flowering phenology of plants may match the regular activity of pollinators, thus increasing the opportunity for pollinator visits and resulting in plant fitness benefits. Non-pollinator agents-mediated directional selection for a greater number of flowers was detected in the HP0N and HP05N treatments. Previous studies have highlighted the potential role of soil drought in shaping the evolution of floral display (Maad and Alexandersson, 2004; Burkle and Runyon, 2016). However, we provided the same amount of water for each individual in the experiment. Soil drought may not be the most important factor driving the evolution of this trait in the present study. In the HP0N and HP05N treatments, non-pollinator agents-mediated disruptive selection on flowering start date was detected. Previous studies have demonstrated the potential role of biotic (e.g., antagonists) and abiotic agents (e.g., temperature, rainfall) in driving the evolution of flowering phenology (Cleland et al., 2007; Elzinga et al., 2007). To clarify which factor contributes to selection on flower production and flowering start date, more manipulative experiments are needed.

The strength of pollinator-mediated selection on four of the six estimated traits first decreased and then increased with increasing nutrient addition intensity. Nutrient availability can affect plant traits, thus influencing pollinator visitation and plant-pollinator interactions (Mattila and Kuitunen, 2000; Burkle and Runyon, 2016; Campbell et al., 2019). Consistent with previous predictions (Brookes et al., 2008; Friberg et al., 2017), our results also demonstrated that N-P-K nutrient availability affected flower production. Flower production in the HP plants, plant reproductive success and pollen limitation intensity covaried non-linearly with increasing nutrient addition intensity. Collectively, these results suggest a non-linear association between the strength of pollinator-mediated directional selection on floral traits and soil N-P-K nutrient availability.

Overall, our results demonstrated that plant traits, plant reproductive success and pollinator-mediated selection on floral traits varied between sites with different soil water contents and among different levels of nutrient addition. Our results support the hypothesis that abiotic environmental factors influence the importance of pollinators in shaping floral evolution.

## Data Availability Statement

Publicly available datasets were analyzed in this study. This data can be found here: https://datadryad.org/stash/share/OSVSSq8HjsxTWFf8Tr92J1MVOHSw8T3I7YIpoq3bmrA.

## Author Contributions

YW and QL conceived and designed the study, analyzed the data, interpreted results, and wrote the manuscript. YW, XD, and ZT conducted the field experiments. All authors contributed to the article and approved the submitted version.

## Conflict of Interest

The authors declare that the research was conducted in the absence of any commercial or financial relationships that could be construed as a potential conflict of interest.

## Publisher’s Note

All claims expressed in this article are solely those of the authors and do not necessarily represent those of their affiliated organizations, or those of the publisher, the editors and the reviewers. Any product that may be evaluated in this article, or claim that may be made by its manufacturer, is not guaranteed or endorsed by the publisher.
