# Corrigendum: Pollinator-Mediated Selection on Floral Traits of *Primula tibetica* Differs Between Sites With Different Soil Water Contents and Among Different Levels of Nutrient Availability

**DOI:** 10.3389/fpls.2022.885464

**Published:** 2022-03-25

**Authors:** Yun Wu, Xuyu Duan, Zhaoli Tong, Qingjun Li

**Affiliations:** ^1^School of Architecture and Civil Engineering, Xihua University, Chengdu, China; ^2^Yunnan Key Laboratory of Plant Reproductive Adaptation and Evolutionary Ecology, Yunnan University, Kunming, China; ^3^Laboratory of Ecology and Evolutionary Biology, School of Ecology and Environmental Science, Yunnan University, Kunming, China; ^4^College of Landscape Architecture, Sichuan Agricultural University, Chengdu, China

**Keywords:** floral evolution, plant-pollinator interactions, pollinator-mediated selection, *Primula tibetica*, soil N-P-K nutrient availability, soil water content, strength of selection

In the original article, there was a mistake in Figure S3 of Supplementary Datasheet 1 as published. The unit of data (mg/kg) in the Y-axis was wrong. The corrected Figure S3 appears below.

**Figure S3:**
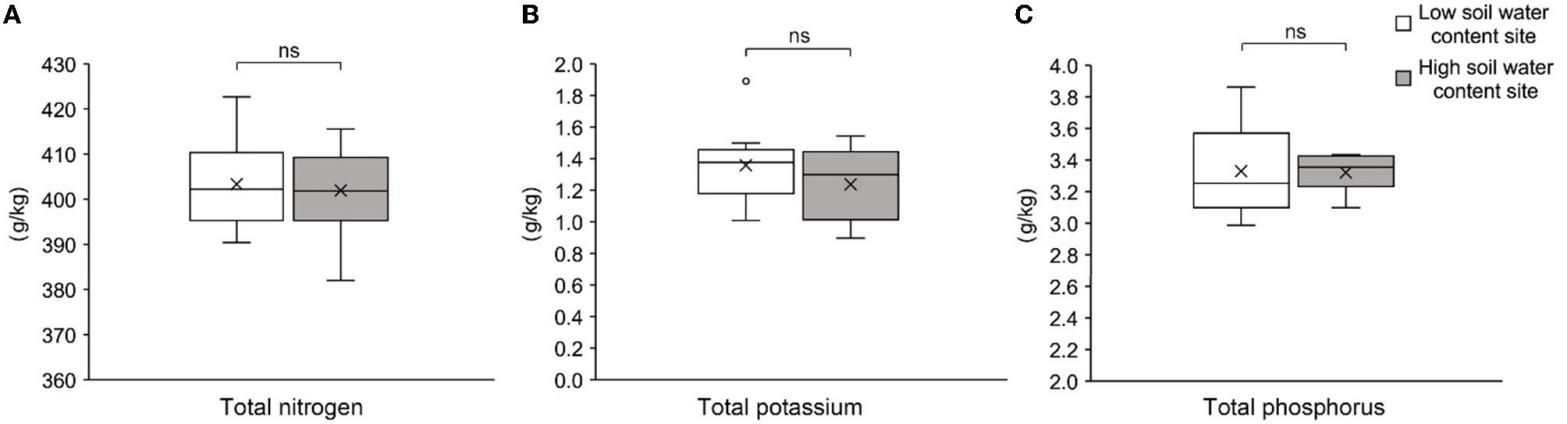


The authors apologize for this error and state that this does not change the scientific conclusions of the article in any way. The original article has been updated.

## Publisher's Note

All claims expressed in this article are solely those of the authors and do not necessarily represent those of their affiliated organizations, or those of the publisher, the editors and the reviewers. Any product that may be evaluated in this article, or claim that may be made by its manufacturer, is not guaranteed or endorsed by the publisher.

