# Supplementary material for: Pollinator-Mediated Selection on Floral Traits of Primula tibetica Differs Between Sites With Different Soil Water Contents and Among Different Levels of Nutrient Availability
Source: Front Plant Sci. 2022 Mar 1;13:807689. doi: 10.3389/fpls.2022.807689 (PMC8921772; doi:10.3389/fpls.2022.807689)
Supplement: Supplementary file 1 [file Data_Sheet_1.pdf]

**Supporting Materials**

**TABLE S1** Exact nutrient concentrations of all nutrients among different nutrient addition treatments.

**Table S2** Directional selection gradients ($\beta_i$ ± SE) and associated *P*-values among open pollination treatment (C) and supplemental hand pollination treatment (HP) at *Primula tibetica* sites in 2019 and 2020.

**TABLE S3** Variations in net directional selection between sites in 2019 and 2020 via ANCOVA testing for *Primula tibetica*.

**TABLE S4** Stabilizing or disruptive selection gradients ($\gamma_{ii}$ ± SE) and associated *P*-values among open pollination treatment (C) and supplemental hand pollination treatment (HP) at *Primula tibetica* sites in 2019 and 2020.

**TABLE S5** Variations in pollinator-mediated directional selection between years and between sites via ANCOVA testing for *Primula tibetica*.

**TABLE S6** Variations in pollinator-mediated stabilizing or disruptive selection between years and between sites via ANCOVA testing for *Primula tibetica*.

**TABLE S7** Variations in pollinator-mediated stabilizing or disruptive selection between sites in 2019 via ANCOVA testing for *Primula tibetica*.

**TABLE S8** Variations in the strength of net selection and pollinator-mediated selection between years and/or between sites via ANCOVA testing for *Primula tibetica.*

**TABLE S9** Directional selection gradients ($\beta_i$ ± SE) and associated *P*-values among the open pollination treatment (C) and supplemental hand pollination treatment (HP) in different N-P-K nutrient addition treatments.

**TABLE S10** Variations in the strength of net selection and pollinator-mediated selection among nutrient addition treatments via ANCOVA testing for *Primula tibetica.*

**TABLE S11** Stabilizing or disruptive selection gradients ($\gamma_{ii}$ ± SE) and associated *P*-values among the open pollination treatment (C) and supplemental hand pollination treatment (HP) in different N-P-K nutrient addition treatments.

**TABLE S12** Pollinator-mediated selection ($\Delta\beta_{poll} \pm$ SE) on floral traits of *Primula tibetica* across different N-P-K nutrient addition treatments.

**TABLE S13** Pollinator-mediated stabilizing or disruptive selection ($\Delta\gamma_{poll} \pm$ SE) on floral traits of *Primula tibetica* across different N-P-K nutrient addition treatments.

**TABLE S14** Variations in net directional selection among nutrient addition treatments via ANCOVA testing for *Primula tibetica*.

**TABLE S15** Variations in pollinator-mediated directional selection among nutrient addition treatments via ANCOVA testing for *Primula tibetica*.

**FIGURE S1** Development of *Primula tibetica* across different soil water availability environments.

**FIGURE S2** Development of *Primula tibetica* across environments with abundant (a) and poor (b) soil nutrient availability.

**FIGURE S3** Boxplot of soil total nitrogen (a), total potassium (b) and total phosphorus (c) between low and high soil water content sites.

**FIGURE S4** Standardized linear phenotypic selection gradients for the plant height in open pollination treatment plants (C, open circles, dashed line) and supplemental hand pollination treatment plants (HP, closed circles, solid line) in 2020 of *Primula tibetica*.

**TABLE S1** Exact nutrient concentrations of all nutrients among different nutrient addition treatments. The N nutrient was applied by both nitrate and ammonium.

| Treatments \ Nutrient types | N (mg) | P (mg) | K (mg) | B (mg) | Zn (mg) | Mn (mg) | Fe (mg) | Cu (mg) | Ca/Mg (mg) |
|---|---|---|---|---|---|---|---|---|---|
| 0.5% 50 ml nutrient addition | 50 | 50 | 50 | 0.25 | 0.25 | 0.125 | 0.325 | 1.25 | 0.5 |
| 1% 50 ml nutrient addition | 100 | 100 | 100 | 0.5 | 0.5 | 0.25 | 0.65 | 2.5 | 1 |
| 2% 50 ml nutrient addition | 200 | 200 | 200 | 1 | 1 | 0.5 | 1.3 | 5 | 2 |

**TABLE S2** Directional selection gradients ($\beta_i$ ± SE) and associated *P*-values among open pollination treatment (C) and supplemental hand pollination treatment (HP) at *Primula tibetica* sites in 2019 and 2020. Pollinator-mediated selection ($\Delta\beta_{poll} = \beta_C - \beta_{HP}$) and *P*-values association with the trait × pollination interactions in ANCOVAs conducted separately for each site and each year are also given. Significant selection estimates and their *P*-values are indicated in bold.

| Traits | C | | HP | | Pollinator-mediated selection | |
|---|---|---|---|---|---|---|
| | $\beta_i$ ± SE | *P* | $\beta_i$ ± SE | *P* | $\Delta\beta_{poll}$ ± SE | *P* |
| Low soil water content site 2019 | | | | | | |
| Flowering duration | 0.053±0.084 | 0.53 | -0.061±0.085 | 0.474 | 0.114±0.120 | 0.672 |
| Plant height | **0.185±0.084** | **0.032** | **0.168±0.074** | **0.026** | 0.017±0.112 | 0.304 |
| Number of flowers | **0.203±0.097** | **0.04** | **0.419±0.084** | **< 0.001** | -0.216±0.128 | 0.076 |
| Corolla size | -0.172±0.112 | 0.129 | 0.063±0.080 | 0.438 | -0.235±0.138 | 0.09 |
| Corolla tube size | 0.140±0.085 | 0.106 | 0.115±0.075 | 0.127 | 0.025±0.113 | 0.508 |
| Corolla tube length | **0.217±0.083** | **0.011** | -0.016±0.071 | 0.821 | 0.233±0.109 | 0.14 |
| High soil water content site 2019 | | | | | | |
| Flowering duration | 0.035±0.058 | 0.552 | 0.013±0.036 | 0.706 | 0.022±0.068 | 0.516 |
| Plant height | -0.026±0.061 | 0.674 | -0.041±0.036 | 0.255 | 0.015±0.071 | 0.665 |
| Number of flowers | **0.474±0.062** | **< 0.001** | **0.507±0.038** | **< 0.001** | -0.033±0.073 | 0.517 |
| Corolla size | 0.098±0.063 | 0.122 | 0.003±0.042 | 0.94 | 0.095±0.076 | 0.208 |
| Corolla tube size | -0.059±0.062 | 0.347 | 0.070±0.039 | 0.077 | -0.129±0.073 | 0.179 |
| Corolla tube length | 0.044±0.056 | 0.436 | 0.029±0.038 | 0.441 | 0.015±0.068 | 0.576 |
| Low soil water content site 2020 | | | | | | |
| Flowering duration | 0.035±0.082 | 0.669 | -0.040±0.044 | 0.368 | 0.075±0.093 | 0.333 |
| Plant height | **0.298±0.088** | **0.001** | 0.063±0.047 | 0.191 | **0.235±0.100** | **< 0.001** |
| Number of flowers | **0.466±0.088** | **< 0.001** | **0.305±0.046** | **< 0.001** | 0.161±0.099 | 0.095 |
| Corolla size | **0.229±0.086** | **0.009** | **0.183±0.054** | **0.001** | 0.046±0.102 | 0.657 |
| Corolla tube size | 0.062±0.083 | 0.455 | -0.044±0.047 | 0.352 | 0.106±0.095 | 0.192 |

| | | | | | | |
|---|---|---|---|---|---|---|
| Corolla tube length | -0.010±0.085 | 0.911 | **0.099±0.049** | **0.048** | -0.109±0.098 | 0.488 |
| High soil water content site 2020 | | | | | | |
| Flowering duration | 0.056±0.062 | 0.375 | 0.006±0.036 | 0.876 | 0.05±0.072 | 0.162 |
| Plant height | 0.092±0.057 | 0.111 | **0.076±0.031** | **0.018** | 0.016±0.065 | 0.159 |
| Number of flowers | **0.380±0.064** | **< 0.001** | **0.408±0.036** | **< 0.001** | -0.028±0.073 | 0.271 |
| Corolla size | **0.142±0.063** | **0.027** | **0.152±0.033** | **< 0.001** | -0.01±0.071 | 0.924 |
| Corolla tube size | 0.057±0.059 | 0.335 | -0.017±0.032 | 0.587 | 0.074±0.067 | 0.481 |
| Corolla tube length | **0.131±0.056** | **0.021** | 0.039±0.032 | 0.224 | 0.092±0.064 | 0.522 |

**TABLE S3** Variations in net directional selection between sites in 2019 and 2020 via ANCOVA testing for *Primula tibetica*.

| Terms | 2019 | | 2020 | |
|---|---|---|---|---|
| | $F_{1,178}$ | $P$ | $F_{1,158}$ | $P$ |
| Site | 0 | 1 | 0 | 1 |
| Flowering duration | 19.032 | < **0.001** | 27.053 | < **0.001** |
| Plant height | 49.316 | < **0.001** | 19.121 | < **0.001** |
| Number of flowers | 93.222 | < **0.001** | 57.932 | < **0.001** |
| Corolla size | 21.499 | < **0.001** | 0.77 | 0.382 |
| Corolla tube size | 0.977 | 0.324 | 0.033 | 0.857 |
| Corolla tube length | 0.961 | 0.328 | 4.43 | **0.037** |
| Site × flowering duration | 0.064 | 0.801 | 0.501 | 0.48 |
| Site × plant height | 11.559 | < **0.001** | 0.093 | 0.761 |
| Site × number of flowers | 0.057 | 0.812 | 6.623 | **0.011** |
| Site × corolla size | 0.314 | 0.576 | 0.725 | 0.396 |
| Site × corolla tube size | 0.027 | 0.869 | 3.444 | 0.066 |
| Site × corolla tube length | 0.589 | 0.444 | 3.113 | 0.08 |

**TABLE S4** Stabilizing or disruptive selection gradients ($\gamma_{ii}$ ± SE) and associated *P*-values among open pollination treatment (C) and supplemental hand pollination treatment (HP) at *Primula tibetica* sites in 2019 and 2020. Pollinator-mediated selection ($\Delta\gamma_{poll} = \gamma_C - \gamma_{HP}$) and *P*-values association with the trait × pollination interactions in ANCOVAs conducted separately for each site and each year are also given. Significant selection estimates and their *P*-values are indicated in bold.

| Traits | C | | HP | | Pollinator-mediated selection | |
|---|---|---|---|---|---|---|
| | $\gamma_{ii}$ ± SE | *P* | $\gamma_{ii}$ ± SE | *P* | $\Delta\gamma_{poll}$ ± SE | *P* |
| Low soil water content site 2019 | | | | | | |
| Flowering duration | 0.017±0.078 | 0.911 | 0.033±0.072 | 0.815 | -0.016±0.106 | 0.479 |
| Plant height | -0.201±0.053 | 0.062 | **0.244±0.050** | **0.017** | **-0.445±0.073** | **0.002** |
| Number of flowers | -0.135±0.040 | 0.099 | **-0.240±0.047** | **0.014** | 0.105±0.062 | 0.672 |
| Corolla size | 0.057±0.065 | 0.664 | 0.118±0.037 | 0.116 | -0.061±0.075 | 0.717 |
| Corolla tube size | -0.019±0.036 | 0.789 | -0.071±0.055 | 0.519 | 0.052±0.066 | 0.694 |
| Corolla tube length | 0.133±0.053 | 0.211 | -0.026±0.052 | 0.806 | 0.159±0.074 | 0.265 |
| High soil water content site 2019 | | | | | | |
| Flowering duration | -0.040±0.038 | 0.607 | -0.022±0.020 | 0.584 | -0.018±0.043 | 0.765 |
| Plant height | 0.013±0.050 | 0.894 | -0.053±0.027 | 0.334 | 0.066±0.057 | 0.605 |
| Number of flowers | 0.096±0.050 | 0.342 | **0.167±0.026** | **0.002** | -0.071±0.056 | 0.379 |
| Corolla size | -0.115±0.050 | 0.251 | 0.008±0.032 | 0.901 | -0.123±0.059 | 0.365 |
| Corolla tube size | 0.086±0.036 | 0.237 | 0.043±0.025 | 0.387 | 0.043±0.044 | 0.738 |
| Corolla tube length | -0.025±0.040 | 0.76 | -0.039±0.025 | 0.447 | 0.014±0.047 | 0.969 |
| Low soil water content site 2020 | | | | | | |
| Flowering duration | 0.068±0.069 | 0.624 | 0.033±0.028 | 0.555 | 0.035±0.074 | 0.655 |
| Plant height | 0.098±0.053 | 0.364 | **0.101±0.022** | **0.025** | **-0.003±0.057** | **0.015** |
| Number of flowers | -0.160±0.066 | 0.232 | **-0.224±0.016** | **< 0.001** | 0.064±0.068 | 0.134 |
| Corolla size | 0.171±0.067 | 0.204 | **0.164±0.028** | **0.005** | 0.007±0.073 | 0.87 |
| Corolla tube size | 0.164±0.066 | 0.217 | 0.051±0.030 | 0.395 | 0.113±0.073 | 0.721 |

| | | | | | | |
|---|---|---|---|---|---|---|
| Corolla tube length | -0.008±0.035 | 0.908 | -0.029±0.029 | 0.623 | 0.021±0.045 | 0.803 |
| High soil water content site 2020 | | | | | | |
| Flowering duration | 0.064±0.044 | 0.464 | -0.007±0.028 | 0.893 | -0.057±0.052 | 0.826 |
| Plant height | 0.104±0.052 | 0.322 | -0.015±0.020 | 0.716 | 0.119±0.056 | 0.397 |
| Number of flowers | -0.053±0.035 | 0.456 | -0.025±0.026 | 0.634 | -0.028±0.044 | 0.603 |
| Corolla size | -0.003±0.044 | 0.976 | 0.077±0.027 | 0.157 | -0.080±0.052 | 0.626 |
| Corolla tube size | -0.028±0.037 | 0.700 | 0.018±0.019 | 0.635 | -0.046±0.042 | 0.696 |
| Corolla tube length | 0.114±0.044 | 0.200 | 0.003±0.020 | 0.949 | 0.111±0.048 | 0.090 |

**TABLE S5** Variations in pollinator-mediated directional selection between years and between sites via ANCOVA testing for *Primula tibetica*.

| Terms | *df* | *F* | *P* |
|---|---|---|---|
| Year | 1 | 0 | 1 |
| Site | 1 | 0 | 1 |
| Pollination | 1 | 0 | 1 |
| Flowering duration | 1 | **96.634** | **< 0.001** |
| Plant height | 1 | **141.255** | **< 0.001** |
| Number of flowers | 1 | **444.167** | **< 0.001** |
| Corolla tube length | 1 | **22.358** | **< 0.001** |
| Corolla tube size | 1 | **11.18** | **< 0.001** |
| Corolla size | 1 | **21.759** | **< 0.001** |
| Year × flowering duration | 1 | 0.195 | 0.659 |
| Year × plant height | 1 | **6.911** | **0.009** |
| Year × number of flowers | 1 | 1.003 | 0.317 |
| Year × corolla tube length | 1 | 1.806 | 0.179 |
| Year × corolla tube size | 1 | 0.02 | 0.888 |
| Year × corolla size | 1 | **11.528** | **< 0.001** |
| Site × flowering duration | 1 | 1.482 | 0.224 |
| Site × plant height | 1 | **12.139** | **< 0.001** |
| Site × number of flowers | 1 | **6.428** | **0.011** |
| Site × corolla tube length | 1 | 0.02 | 0.887 |
| Site × corolla tube size | 1 | 1.307 | 0.253 |
| Site × corolla size | 1 | 0.02 | 0.888 |
| Pollination × flowering duration | 1 | 1.664 | 0.198 |
| Pollination × plant height | 1 | 0.316 | 0.574 |
| Pollination × number of flowers | 1 | 0.313 | 0.576 |
| Pollination × corolla tube length | 1 | 0.863 | 0.353 |
| Pollination × corolla tube size | 1 | 0.044 | 0.834 |
| Pollination × corolla size | 1 | 0.009 | 0.926 |
| Year × site × flowering duration | 1 | 0.705 | 0.401 |
| Year × site × plant height | 1 | 0.342 | 0.559 |
| Year × site × number of flowers | 1 | 2.684 | 0.102 |
| Year × site × corolla tube length | 1 | 0.351 | 0.554 |
| Year × site × corolla tube size | 1 | 0.822 | 0.365 |
| Year × site × corolla size | 1 | 2.39 | 0.123 |
| Year × pollination × flowering duration | 1 | 1.553 | 0.213 |
| Year × pollination × plant height | 1 | 1.859 | 0.173 |
| Year × pollination × number of flowers | 1 | **8.069** | **0.005** |
| Year × pollination × corolla tube length | 1 | 0.669 | 0.414 |
| Year × pollination × corolla tube size | 1 | **4.748** | **0.03** |
| Year × pollination × corolla size | 1 | 0.362 | 0.548 |
| Site × pollination × flowering duration | 1 | 0.009 | 0.926 |

| | | | |
|---|---|---|---|
| Site × pollination × plant height | 1 | 2.589 | 0.108 |
| Site × pollination × number of flowers | 1 | 0.158 | 0.692 |
| Site × pollination × corolla tube length | 1 | 0.006 | 0.939 |
| Site × pollination × corolla tube size | 1 | 0.043 | 0.836 |
| Site × pollination × corolla size | 1 | 1.29 | 0.256 |
| Year × site × pollination × flowering duration | 1 | 0.596 | 0.44 |
| Year × site × pollination × plant height | 1 | **8.321** | **0.004** |
| Year × site × pollination × number of flowers | 1 | 2.169 | 0.141 |
| Year × site × pollination × corolla tube length | 1 | 2.35 | 0.126 |
| Year × site × pollination × corolla tube size | 1 | 0.042 | 0.839 |
| Year × site × pollination × corolla size | 1 | 3.568 | 0.059 |
| Residuals | 628 | | |

**TABLE S6** Variations in pollinator-mediated stabilizing or disruptive selection between years and between sites via ANCOVA testing for *Primula tibetica*. The superscripts indicated the quadratic terms of the standardized floral traits.

| Terms | *df* | *F* | *P* |
|---|---|---|---|
| Year | 1 | 0 | 1 |
| Site | 1 | 0 | 1 |
| Pollination | 1 | 0 | 1 |
| Flowering duration | 1 | **99.249** | **< 0.001** |
| Plant height | 1 | **145.078** | **< 0.001** |
| Number of flowers | 1 | **456.186** | **< 0.001** |
| Corolla tube length | 1 | **22.963** | **< 0.001** |
| Corolla tube size | 1 | **11.482** | **< 0.001** |
| Corolla size | 1 | **22.348** | **< 0.001** |
| (Flowering duration)$^2$ | 1 | 0.214 | 0.643 |
| (Plant height)$^2$ | 1 | **6.285** | **0.012** |
| (Number of flowers)$^2$ | 1 | **7.157** | **0.008** |
| (Corolla tube length)$^2$ | 1 | 0.042 | 0.837 |
| (Corolla tube size)$^2$ | 1 | 2.52 | 0.113 |
| (Corolla size)$^2$ | 1 | **5.046** | **0.025** |
| Year × (Flowering duration)$^2$ | 1 | 0.662 | 0.416 |
| Year × (Plant height)$^2$ | 1 | **6.547** | **0.011** |
| Year × (Number of flowers)$^2$ | 1 | 1.015 | 0.314 |
| Year × (Corolla tube length)$^2$ | 1 | 1.138 | 0.287 |
| Year × (Corolla tube size)$^2$ | 1 | 0.029 | 0.865 |
| Year × (Corolla size)$^2$ | 1 | 1.183 | 0.277 |
| Site × (Flowering duration)$^2$ | 1 | 0.359 | 0.549 |
| Site × (Plant height)$^2$ | 1 | **9.794** | **0.002** |
| Site × (Number of flowers)$^2$ | 1 | **37.307** | **< 0.001** |
| Site × (Corolla tube length)$^2$ | 1 | 0.125 | 0.723 |
| Site × (Corolla tube size)$^2$ | 1 | 0.008 | 0.927 |
| Site × (Corolla size)$^2$ | 1 | 3.84 | 0.051 |
| Pollination × (Flowering duration)$^2$ | 1 | 0.025 | 0.874 |
| Pollination × (Plant height)$^2$ | 1 | 0.101 | 0.751 |
| Pollination × (Number of flowers)$^2$ | 1 | 1.984 | 0.159 |
| Pollination × (Corolla tube length)$^2$ | 1 | 0.526 | 0.468 |
| Pollination × (Corolla tube size)$^2$ | 1 | 0.033 | 0.856 |
| Pollination × (Corolla size)$^2$ | 1 | 0.283 | 0.595 |
| Year × site × (Flowering duration)$^2$ | 1 | 0.002 | 0.967 |
| Year × site × (Plant height)$^2$ | 1 | 0.656 | 0.418 |
| Year × site × (Number of flowers)$^2$ | 1 | 2.564 | 0.11 |
| Year × site × (Corolla tube length)$^2$ | 1 | 1.625 | 0.203 |
| Year × site × (Corolla tube size)$^2$ | 1 | 0.808 | 0.369 |
| Year × site × (Corolla size)$^2$ | 1 | 0.427 | 0.514 |

| | | | |
|---|---|---|---|
| Year × pollination × (Flowering duration)$^2$ | 1 | 1.007 | 0.316 |
| Year × pollination × (Plant height)$^2$ | 1 | **5.331** | **0.021** |
| Year × pollination × (Number of flowers)$^2$ | 1 | 2.736 | 0.099 |
| Year × pollination × (Corolla tube length)$^2$ | 1 | 2.476 | 0.116 |
| Year × pollination × (Corolla tube size)$^2$ | 1 | 1.115 | 0.291 |
| Year × pollination × (Corolla size)$^2$ | 1 | 0.171 | 0.679 |
| Site × pollination × (Flowering duration)$^2$ | 1 | 0.022 | 0.883 |
| Site × pollination × (Plant height)$^2$ | 1 | 0.052 | 0.82 |
| Site × pollination × (Number of flowers)$^2$ | 1 | 0.119 | 0.73 |
| Site × pollination × (Corolla tube length)$^2$ | 1 | 0.094 | 0.759 |
| Site × pollination × (Corolla tube size)$^2$ | 1 | 0.301 | 0.583 |
| Site × pollination × (Corolla size)$^2$ | 1 | 0.293 | 0.588 |
| Year × site × pollination × (Flowering duration)$^2$ | 1 | 0.04 | 0.842 |
| Year × site × pollination × (Plant height)$^2$ | 1 | **9.865** | **0.002** |
| Year × site × pollination × (Number of flowers)$^2$ | 1 | 0.017 | 0.896 |
| Year × site × pollination × (Corolla tube length)$^2$ | 1 | 1.024 | 0.312 |
| Year × site × pollination × (Corolla tube size)$^2$ | 1 | 0.012 | 0.912 |
| Year × site × pollination × (Corolla size)$^2$ | 1 | 0.257 | 0.613 |
| Residuals | 622 | | |

**TABLE S7** Variations in pollinator-mediated stabilizing or disruptive selection between sites in 2019 via ANCOVA testing for *Primula tibetica*. The superscripts indicated the quadratic terms of the standardized floral traits.

| Terms | *df* | *F* | *P* |
|---|---|---|---|
| Site | 1 | 0 | 1 |
| Pollination | 1 | 0 | 1 |
| Flowering duration | 1 | **65.009** | **< 0.001** |
| Plant height | 1 | **38.047** | **< 0.001** |
| Number of flowers | 1 | **219.485** | **< 0.001** |
| Corolla tube length | 1 | **4.669** | **0.032** |
| Corolla tube size | 1 | **7.702** | **0.006** |
| Corolla size | 1 | 0.234 | 0.629 |
| (Flowering duration)$^2$ | 1 | 0.765 | 0.383 |
| (Plant height)$^2$ | 1 | 0.574 | 0.449 |
| (Number of flowers)$^2$ | 1 | 3.238 | 0.073 |
| (Corolla tube length)$^2$ | 1 | 0.33 | 0.566 |
| (Corolla tube size)$^2$ | 1 | 2.648 | 0.105 |
| (Corolla size)$^2$ | 1 | 1.82 | 0.178 |
| Site × (Flowering duration)$^2$ | 1 | 0.005 | 0.946 |
| Site × (Plant height)$^2$ | 1 | 1.264 | 0.262 |
| Site × (Number of flowers)$^2$ | 1 | **23.99** | **< 0.001** |
| Site × (Corolla tube length)$^2$ | 1 | 0.649 | 0.421 |
| Site × (Corolla tube size)$^2$ | 1 | 0.305 | 0.581 |
| Site × (Corolla size)$^2$ | 1 | **4.305** | **0.039** |
| Pollination × (Flowering duration)$^2$ | 1 | 0.473 | 0.492 |
| Pollination × (Plant height)$^2$ | 1 | **4.124** | **0.043** |
| Pollination × (Number of flowers)$^2$ | 1 | 0.296 | 0.587 |
| Pollination × (Corolla tube length)$^2$ | 1 | 1.218 | 0.271 |
| Pollination × (Corolla tube size)$^2$ | 1 | 0.59 | 0.443 |
| Pollination × (Corolla size)$^2$ | 1 | 1.158 | 0.283 |
| Site × pollination × (Flowering duration)$^2$ | 1 | 0.063 | 0.802 |
| Site × pollination × (Plant height)$^2$ | 1 | **6.497** | **0.011** |
| Site × pollination × (Number of flowers)$^2$ | 1 | 0.429 | 0.513 |
| Site × pollination × (Corolla tube length)$^2$ | 1 | 0.937 | 0.334 |
| Site × pollination × (Corolla tube size)$^2$ | 1 | 0.55 | 0.459 |
| Site × pollination × (Corolla size)$^2$ | 1 | 0.356 | 0.551 |
| Residuals | 293 | | |

**TABLE S8** Variations in the strength of net selection and pollinator-mediated selection between years and/or between sites via ANCOVA testing for *Primula tibetica.*

| Terms | Strength of net directional selection | | Strength of net stabilizing or disruptive selection | | Strength of pollinator-mediated directional selection | | Strength of pollinator-mediated stabilizing or disruptive selection | |
|---|---|---|---|---|---|---|---|---|
| | $F_{1,23}$ | $P$ | $F_{1,23}$ | $P$ | $F_{1,23}$ | $P$ | $F_{1,23}$ | $P$ |
| Year | 0.131 | 0.721 | 0.119 | 0.733 | 0.195 | 0.664 | 1.351 | 0.259 |
| Site | 0.466 | 0.502 | 2.987 | 0.099 | **8.894** | **0.007** | 0.525 | 0.477 |
| Year × site | < 0.001 | 0.991 | 0.167 | 0.687 | 0.043 | 0.838 | 2.776 | 0.111 |

**TABLE S9** Directional selection gradients ($\beta_i \pm$ SE) and associated *P*-values among the open pollination treatment (C) and supplemental hand pollination treatment (HP) in different N-P-K nutrient addition treatments. Significant selection estimates and their *P*-values are indicated in bold.

| Traits | 0% nutrient addition-C | | 0% nutrient addition-HP | | 0.5% nutrient addition-C | | 0.5% nutrient addition-HP | | 1% nutrient addition-C | | 1% nutrient addition-HP | |
|---|---|---|---|---|---|---|---|---|---|---|---|---|
| | $\beta_i \pm$ SE | *P* | $\beta_i \pm$ SE | *P* | $\beta_i \pm$ SE | *P* | $\beta_i \pm$ SE | *P* | $\beta_i \pm$ SE | *P* | $\beta_i \pm$ SE | *P* |
| Flowering start date | **-0.283±0.118** | **0.02** | -0.153±0.082 | 0.068 | -0.111±0.092 | 0.236 | -0.084±0.091 | 0.358 | -0.212±0.178 | 0.239 | -0.103±0.180 | 0.57 |
| Flowering duration | -0.012±0.150 | 0.937 | 0.030±0.099 | 0.761 | 0.023±0.145 | 0.875 | 0.065±0.112 | 0.564 | 0.179±0.197 | 0.37 | 0.009±0.195 | 0.963 |
| Plant height | **-0.204±0.101** | **0.048** | -0.084±0.071 | 0.24 | 0.026±0.073 | 0.723 | 0.035±0.088 | 0.692 | -0.234±0.156 | 0.142 | 0.055±0.147 | 0.709 |
| Number of flowers | **0.332±0.136** | **0.018** | **0.434±0.101** | **< 0.001** | **0.483±0.120** | **< 0.001** | **0.379±0.099** | **< 0.001** | 0.308±0.189 | 0.11 | 0.191±0.205 | 0.355 |
| Corolla tube size | **0.216±0.102** | **0.039** | 0.014±0.071 | 0.844 | 0.033±0.078 | 0.676 | 0.056±0.092 | 0.546 | -0.124±0.172 | 0.473 | -0.155±0.158 | 0.332 |
| Corolla tube length | -0.098±0.111 | 0.379 | 0.034±0.066 | 0.613 | 0.051±0.075 | 0.5 | 0.052±0.081 | 0.52 | -0.157±0.167 | 0.352 | 0.076±0.142 | 0.593 |

**TABLE S10** Variations in the strength of net selection and pollinator-mediated selection among nutrient addition treatments via ANCOVA testing for *Primula tibetica*.

| Terms | Strength of net directional selection | | Strength of net stabilizing or disruptive selection | | Strength of pollinator-mediated directional selection | | Strength of pollinator-mediated stabilizing or disruptive selection | |
|---|---|---|---|---|---|---|---|---|
| | $F_{2,17}$ | $P$ | $F_{2,17}$ | $P$ | $F_{2,17}$ | $P$ | $F_{2,17}$ | $P$ |
| Nutrient addition | 0.686 | 0.519 | 0.546 | 0.591 | **5.634** | **0.015** | 0.581 | 0.571 |

**TABLE S11** Stabilizing or disruptive selection gradients ($\gamma_{ii}$ ± SE) and associated *P*-values among the open pollination treatment (C) and supplemental hand pollination treatment (HP) in different N-P-K nutrient addition treatments. Significant selection estimates and their *P*-values are indicated in bold.

| Traits | 0% nutrient addition-C | | 0% nutrient addition-HP | | 0.5% nutrient addition-C | | 0.5% nutrient addition-HP | | 1% nutrient addition-C | | 1% nutrient addition-HP | |
|---|---|---|---|---|---|---|---|---|---|---|---|---|
| | $\gamma_{ii}$ ± SE | *P* | $\gamma_{ii}$ ± SE | *P* | $\gamma_{ii}$ ± SE | *P* | $\gamma_{ii}$ ± SE | *P* | $\gamma_{ii}$ ± SE | *P* | $\gamma_{ii}$ ± SE | *P* |
| Flowering start date | 0.044±0.076 | 0.775 | **0.294±0.064** | **0.026** | -0.131±0.070 | 0.359 | **0.530±0.081** | **0.002** | **1.023±0.173** | **0.005** | -0.049±0.157 | 0.878 |
| Flowering duration | 0.084±0.099 | 0.674 | -0.013±0.069 | 0.926 | -0.138±0.086 | 0.428 | 0.039±0.069 | 0.779 | -0.008±0.154 | 0.979 | 0.141±0.170 | 0.679 |
| Plant height | -0.073±0.102 | 0.723 | -0.071±0.054 | 0.514 | -0.128±0.042 | 0.137 | 0.059±0.076 | 0.7 | 0.138±0.082 | 0.407 | 0.309±0.158 | 0.332 |
| Number of flowers | -0.221±0.092 | 0.234 | -0.038±0.071 | 0.787 | 0.284±0.078 | 0.079 | 0.080±0.067 | 0.554 | -0.084±0.127 | 0.743 | 0.126±0.200 | 0.754 |
| Corolla tube size | -0.239±0.073 | 0.107 | 0.037±0.055 | 0.739 | 0.103±0.050 | 0.306 | 0.172±0.055 | 0.123 | -0.151±0.067 | 0.27 | 0.027±0.128 | 0.916 |
| Corolla tube length | 0.056±0.074 | 0.709 | 0.024±0.054 | 0.821 | 0.101±0.064 | 0.433 | -0.080±0.051 | 0.434 | -0.101±0.088 | 0.569 | -0.084±0.110 | 0.705 |

**TABLE S12** Pollinator-mediated selection ($\Delta\beta_{poll}$ ± SE) on floral traits of *Primula tibetica* across different N-P-K nutrient addition treatments. Pollinator-mediated selection ($\Delta\beta_{poll} = \beta_C - \beta_{HP}$) and *P*-values association with the trait × pollination interactions in ANCOVAs conducted separately for each treatment are also given.

| Traits | 0% nutrient addition | | 0.5% nutrient addition | | 1% nutrient addition | |
|---|---|---|---|---|---|---|
| | $\Delta\beta_{poll}$ ± SE | *P* | $\Delta\beta_{poll}$ ± SE | *P* | $\Delta\beta_{poll}$ ± SE | *P* |
| Flowering start date | -0.130±0.144 | 0.904 | -0.027±0.129 | 0.746 | -0.109±0.253 | 0.479 |
| Flowering duration | -0.042±0.180 | 0.303 | -0.042±0.183 | 0.728 | 0.170±0.277 | 0.409 |
| Plant height | -0.120±0.123 | 0.205 | -0.009±0.114 | 0.93 | -0.289±0.214 | 0.193 |
| Number of flowers | -0.102±0.169 | 0.568 | 0.104±0.156 | 0.542 | 0.117±0.279 | 0.675 |
| Corolla tube size | 0.212±0.124 | 0.193 | -0.023±0.121 | 0.91 | 0.031±0.234 | 0.91 |
| Corolla tube length | -0.132±0.129 | 0.271 | -0.001±0.110 | 0.951 | -0.233±0.219 | 0.291 |

**TABLE S13** Pollinator-mediated stabilizing or disruptive selection ($\Delta\gamma_{poll}$ ± SE) on floral traits of *Primula tibetica* across different N-P-K nutrient addition treatments. Pollinator-mediated selection ($\Delta\gamma_{poll} = \gamma_C - \gamma_{HP}$) and *P*-values association with the trait × pollination interactions in ANCOVAs conducted separately for each treatment are also given.

| Traits | 0% nutrient addition | | 0.5% nutrient addition | | 1% nutrient addition | |
|---|---|---|---|---|---|---|
| | $\Delta\gamma_{poll}$ ± SE | *P* | $\Delta\gamma_{poll}$ ± SE | *P* | $\Delta\gamma_{poll}$ ± SE | *P* |
| Flowering start date | -0.250±0.099 | 0.273 | **-0.661±0.107** | **0.004** | 1.072±0.234 | 0.057 |
| Flowering duration | 0.097±0.121 | 0.648 | -0.177±0.110 | 0.834 | -0.149±0.229 | 0.281 |
| Plant height | -0.002±0.115 | 0.283 | -0.187±0.087 | 0.431 | -0.171±0.178 | 0.342 |
| Number of flowers | -0.183±0.116 | 0.347 | 0.204±0.103 | 0.39 | -0.210±0.237 | 0.421 |
| Corolla tube size | -0.276±0.091 | 0.208 | -0.069±0.074 | 0.479 | -0.178±0.144 | 0.739 |
| Corolla tube length | 0.032±0.092 | 0.928 | 0.181±0.082 | 0.298 | -0.017±0.141 | 0.913 |

**TABLE S14** Variations in net directional selection among nutrient addition treatments via ANCOVA testing for *Primula tibetica*.

| Terms | *df* | *F* | *P* |
|---|---|---|---|
| Nutrient addition | 2 | 0 | 1 |
| Flowering start date | 1 | **21.589** | **< 0.001** |
| Flowering duration | 1 | **14.298** | **< 0.001** |
| Plant height | 1 | **5.953** | **0.016** |
| Corolla tube size | 1 | 0.753 | 0.387 |
| Corolla tube length | 1 | 0.17 | 0.68 |
| Number of flowers | 1 | **14.478** | **< 0.001** |
| Nutrient addition × flowering start date | 2 | 0.109 | 0.896 |
| Nutrient addition × flowering duration | 2 | 0.789 | 0.456 |
| Nutrient addition × plant height | 2 | 0.923 | 0.399 |
| Nutrient addition × corolla tube size | 2 | 2.642 | 0.074 |
| Nutrient addition × corolla tube length | 2 | 0.808 | 0.448 |
| Nutrient addition × number of flowers | 2 | 0.236 | 0.79 |
| Residuals | 152 | | |

**TABLE S15** Variations in pollinator-mediated directional selection among nutrient addition treatments via ANCOVA testing for *Primula tibetica*.

| Terms | *df* | *F* | *P* |
|---|---|---|---|
| Nutrient addition | 2 | 0 | 1 |
| Pollination | 1 | 0 | 1 |
| Flowering start date | 1 | **46.886** | **< 0.001** |
| Flowering duration | 1 | **29.606** | **< 0.001** |
| Plant height | 1 | 3.42 | 0.065 |
| Corolla tube size | 1 | 0.362 | 0.548 |
| Corolla tube length | 1 | 0.177 | 0.674 |
| Number of flowers | 1 | **34.026** | **< 0.001** |
| Nutrient addition × flowering start date | 2 | 0.366 | 0.694 |
| Nutrient addition × flowering duration | 2 | 0.821 | 0.441 |
| Nutrient addition × plant height | 2 | 1.207 | 0.301 |
| Nutrient addition × corolla tube size | 2 | **3.033** | **0.05** |
| Nutrient addition × corolla tube length | 2 | 0.361 | 0.697 |
| Nutrient addition × number of flowers | 2 | 0.959 | 0.384 |
| Pollination × flowering start date | 1 | 0.499 | 0.48 |
| Pollination × flowering duration | 1 | 0.041 | 0.839 |
| Pollination × plant height | 1 | 3.217 | 0.074 |
| Pollination × corolla tube size | 1 | 0.298 | 0.586 |
| Pollination × corolla tube length | 1 | 1.97 | 0.161 |
| Pollination × number of flowers | 1 | 0.036 | 0.85 |
| Nutrient addition × pollination × flowering start date | 2 | 0.327 | 0.722 |
| Nutrient addition × pollination × flowering duration | 2 | 0.883 | 0.415 |
| Nutrient addition × pollination × plant height | 2 | 0.717 | 0.489 |
| Nutrient addition × pollination × corolla tube size | 2 | 0.419 | 0.658 |
| Nutrient addition × pollination × corolla tube length | 2 | 0.541 | 0.582 |
| Nutrient addition × pollination × number of flowers | 2 | 0.34 | 0.712 |
| Residuals | 318 | | |

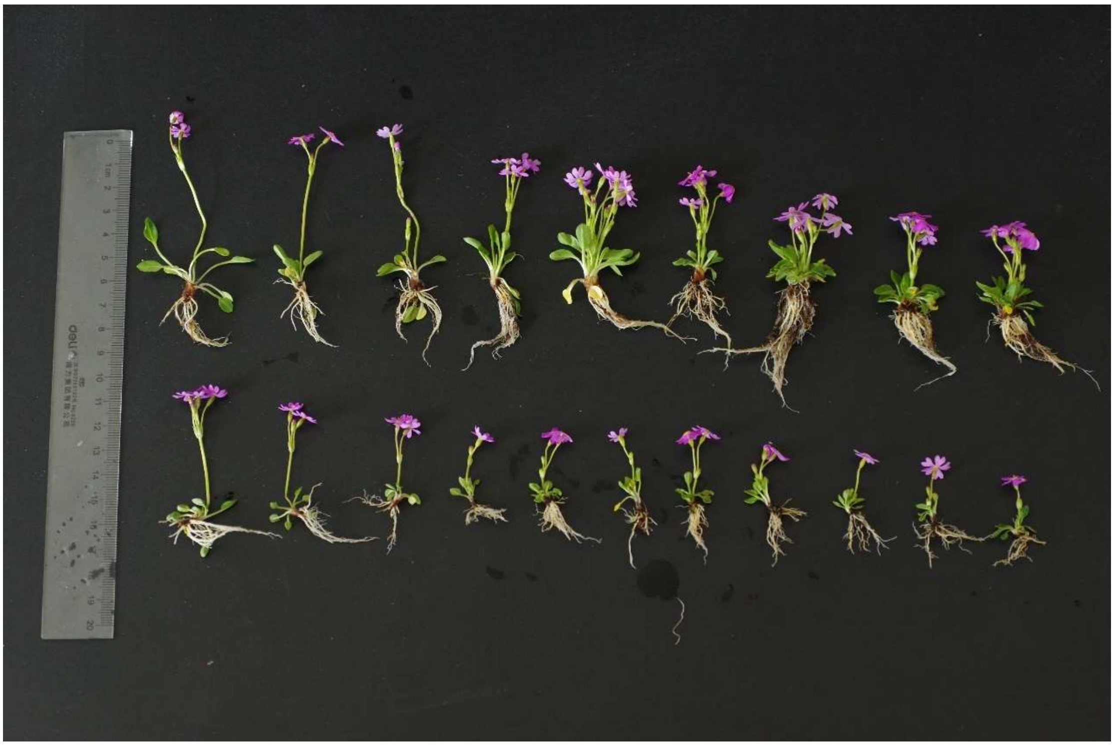

**FIGURE S1** Development of *Primula tibetica* across different soil water availability environments. The upper individuals are from high soil water content environment and the lower individuals are from low soil water content environment.

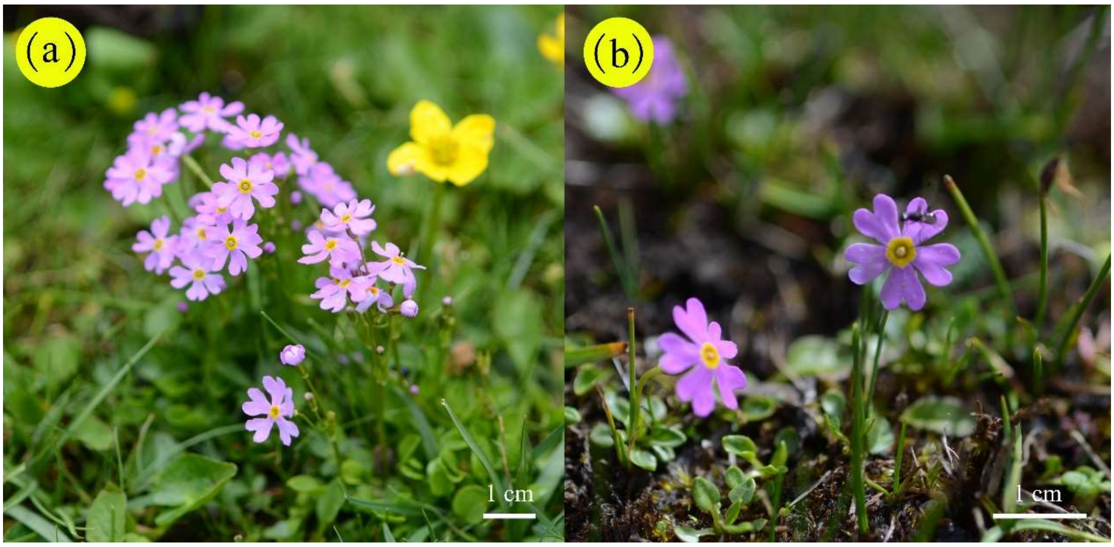


**FIGURE S2** Development of *Primula tibetica* across environments with abundant (a) and poor (b) soil nutrient availability.

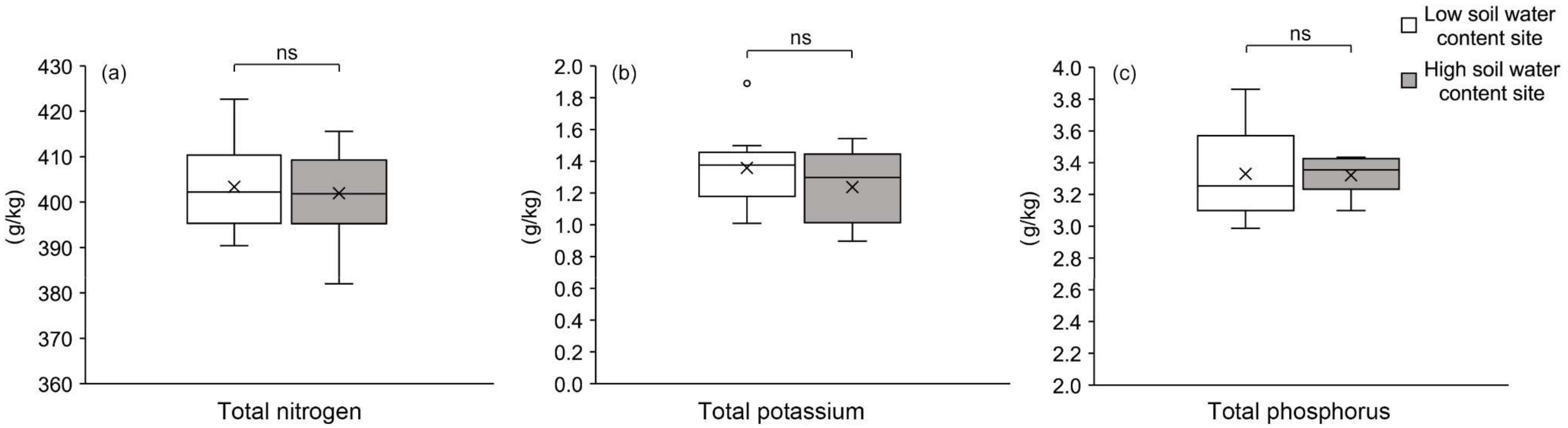


**FIGURE S3** Boxplot of soil total nitrogen (a), total potassium (b) and total phosphorus (c) between low and high soil water content sites. Sample sizes were 20. The '×' was the mean values and the line next to the mean values was the median values. The symbols above the lines indicated the level of significant. ns $P > 0.1$.

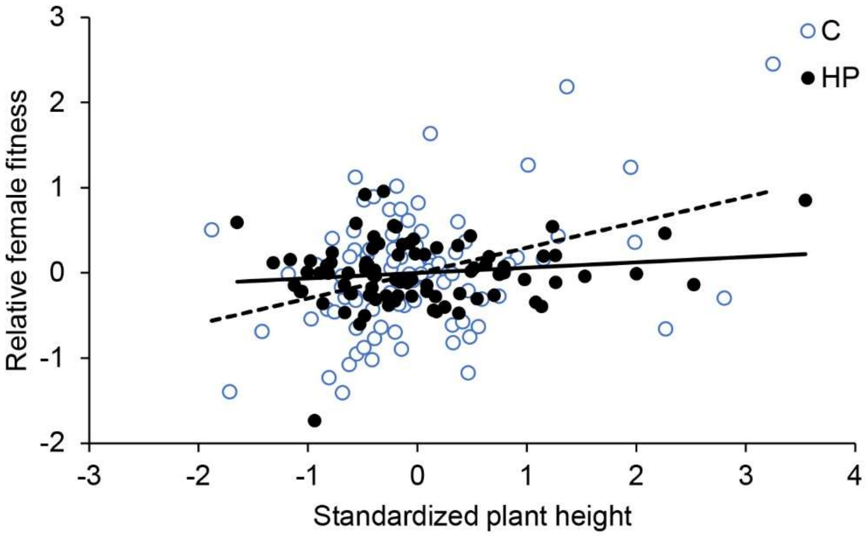


**FIGURE S4** Standardized linear phenotypic selection gradients for the plant height in open pollination treatment plants (C, open circles, dashed line) and supplemental hand pollination treatment plants (HP, closed circles, solid line) in 2020 of *Primula tibetica*. The selection gradients are illustrated with added-variable plot, in which the residuals from a linear regression model of relative seeds per plant on all traits except the focal trait are plotted against the residuals from a regression model of the focal trait on the other traits.
